# Sensors of Infection: Viral Nucleic Acid PRRs in Fish

**DOI:** 10.3390/biology4030460

**Published:** 2015-07-08

**Authors:** Sarah Poynter, Graeme Lisser, Andrea Monjo, Stephanie DeWitte-Orr

**Affiliations:** 1 Department of Biology, Wilfrid Laurier University, 75 University Ave W, Waterloo, ON N2L 3C5, Canada; E-Mails: poyn7090@mylaurier.ca (S.P.); liss8300@mylaurier.ca (G.L.); monj6630@mylaurier.ca (A.M.); 2 Department of Biology, University of Waterloo, 200 University Ave W, Waterloo, ON N2L 3G1, Canada

**Keywords:** pattern recognition receptors, fish, nucleic acids, dsRNA, PAMPs, type I interferon, inflammation, toll-like receptors, RIG-I, SR-A

## Abstract

Viruses produce nucleic acids during their replication, either during genomic replication or transcription. These nucleic acids are present in the cytoplasm or endosome of an infected cell, or in the extracellular space to be sensed by neighboring cells during lytic infections. Cells have mechanisms of sensing virus-generated nucleic acids; these nucleic acids act as flags to the cell, indicating an infection requiring defense mechanisms. The viral nucleic acids are called pathogen-associated molecular patterns (PAMPs) and the sensors that bind them are called pattern recognition receptors (PRRs). This review article focuses on the most recent findings regarding nucleic acids PRRs in fish, including: Toll-like receptors (TLRs), RIG-I-like receptors (RLRs), cytoplasmic DNA sensors (CDSs) and class A scavenger receptors (SR-As). It also discusses what is currently known of the downstream signaling molecules for each PRR family and the resulting antiviral response, either type I interferons (IFNs) or pro-inflammatory cytokine production. The review highlights what is known but also defines what still requires elucidation in this economically important animal. Understanding innate immune systems to virus infections will aid in the development of better antiviral therapies and vaccines for the future.

## 1. Introduction

**Figure 1:**
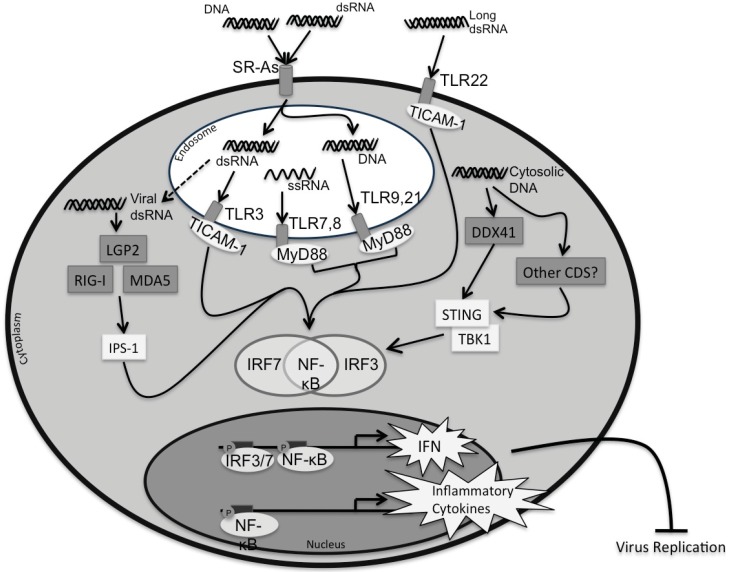
Fish nucleic acid PRR signaling pathways. The cell possesses a number of PRRs capable of sensing nucleic acids (DNA, dsRNA, ssRNA), which can be found on the cell surface, in endosomes, or the cytosol. On the cell surface, the fish-specific TLR22 can sense long extracellular dsRNA molecules and recruit and activate the adaptor protein TICAM-1. Surface SR-As bind nucleic acids (such as DNA and dsRNA) in the extracellular space and transport these nucleic acids into endosomes. Endosomal nucleic acid PRRs include TLR3, which recognizes dsRNA and recruits the adaptor protein TICAM-1. TLR7 and TLR8 are also located in the endosome, where they recognize ssRNA and recruit the adaptor protein MyD88. Lastly, TLR9 and TLR21, located in the endosome, recognize DNA and, like TLR7,8, recruit MyD88 for signaling. With respect to cytosolic nucleic acids, LGP2, RIG-I, and MDA5 (collectively referred to as RLRs) all recognize viral dsRNA in the cytosol and activate IPS-1 for downstream signaling. Endosomal dsRNA can escape (through a yet unknown mechanism), which may be recognized by cytosolic RLRs to activate the downstream signalling pathway via IPS-1 (indicated by a dashed arrow). Cytosolic DNA, on other hand, is recognized by the CDSs. In fish, DDX41 remains the only CDS described thus far. DDX41 activation leads to the recruitment and activation of the adaptor protein STING and subsequent activation of the kinase TBK1. Whether additional CDSs exist is fish is an area of ongoing research. Once the nucleic acid PRRs recognize their specific ligands, their associated adaptor proteins signal through a series of intracellular kinases (not shown) to phosphorylate one, or a combination of, transcription factors (IRF3, 7, and NF-κB). Once activated, these transcription factors translocate to the nucleus and bind to their corresponding regulatory domains to induce the expression of type I IFN and pro-inflammatory cytokines, which together trigger antiviral functions within the host. CDS = cytosolic DNA sensor; CpG DNA = cytosine-phosphate-guanosine deoxyribonucleic acids (DNA); dsRNA = double-stranded ribonucleic acids (RNA); IFN = type I interferon; IKK = IκB kinase; IPS-1 = IFN-β promoter stimulator 1; IRF = interferon regulatory factor; LGP2 = laboratory of genetics and physiology 2; MDA5 = melanoma differentiation-associated gene 5; MyD88 = myeloid differentiation primary response protein 88; NF-κB = neural factor κB; RIG-I = retinoic acid-inducible gene I; single-stranded RNA = ssRNA; STING = stimulator of interferon genes; SR-A = class A scavenger receptor; TBKI = tank-binding kinase-1; TICAM-1 = toll-like receptor adaptor molecule 1; TLR = toll-like receptor.

Nucleic acids, both RNA and DNA, are potent inducers of the innate immune system in vertebrates. Innate immunity is based on the detection of pathogen-associated molecular patterns (PAMPs) by host-derived pattern recognition receptors (PRRs). Under homeostatic cell conditions, genomic DNA is contained within the nucleus and single-stranded mRNA is transported between the nucleus and cytoplasm for protein translation. During virus replication, foreign nucleic acids are produced in either an atypical cellular compartment (*ex.* dsRNA or DNA in the cytoplasm, nucleic acids in endosomes) or with a unique sequence, length, or molecular modification that differentiate them from endogenous nucleic acids [1]. Host PRRs, which activate signaling pathways to mount responses that protect the cell, recognize these foreign nucleic acids (PAMPs). The three types of nucleic acids produced during virus infections are: single-stranded (ss)RNA, double-stranded (ds)RNA, and DNA. Host ssRNA molecules contain a 5' cap and 3' poly A tail to aid in stability and transport within the cell. ssRNA molecules present during virus replication, either as viral genomes or transcripts, contain guanosine- and uridine-rich sequences [2] or 5' triphosphates [3] that label them as PAMPs. dsRNA molecules are produced by virtually all viruses at some point during their replication cycle; they can originate from dsRNA virus genome fragments, from ssRNA virus replicative intermediates, or as byproducts of DNA virus bidirectional convergent transcription [4]. The dsRNA helix is structurally unique compared to dsDNA and it is this structure, combined with a minimum length of 30 bp that most PRRs use to sense dsRNA as a PAMP. Healthy host cells do make dsRNA molecules for the RNAi pathway, used to control gene expression and cellular homeostasis; however, these molecules are under 30 bp in vertebrates to avoid PRR detection and the IFN response [5]. Moreover, DNA is also detected by several PRRs. Genomic DNA from DNA viruses can be released into the cytoplasm by proteasomal degradation of their capsid [6]. DNA can then be sensed by cytosolic DNA PRRs and can also be transcribed into RNA by RNA polymerase III, which enables sensing by cytosolic RNA sensors as well [7]. These nucleic acid PRRs trigger signaling cascades, which culminate in the induction of either type I interferon (IFN) or inflammasome-mediated inflammatory responses. This review provides an in-depth look at the current literature regarding the families of PRRs that sense viral nucleic acids within teleost fish cells, including toll-like receptors (TLRs), RIG-I-like receptors (RLRs), cytosolic DNA sensors (CDSs), and class A scavenger receptors (SR-As), while also discussing their downstream signaling pathways and roles in the teleost antiviral response. A summary of the known nucleic acid PRRs in fish, their cellular location, and downstream signaling pathways are summarized in Figure 1.

## 2. Pattern Recognition Receptors (PRRs)

**Figure 2:**
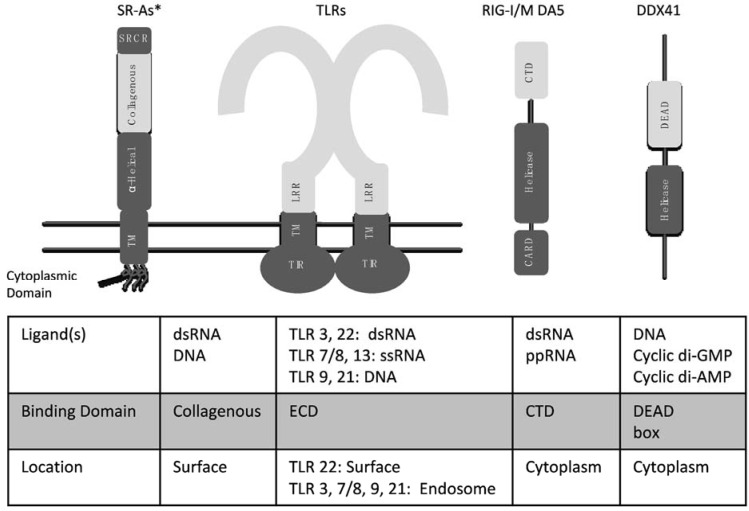
Summary of structural domains found in fish nucleic acid sensors involved in antiviral immunity. The key structural domains are labeled for each nucleic acid PRR family, with the nucleic acid binding region indicated by lighter shading within the structure. The ligands, binding domains, and cellular location for each PRR are listed in the table below. * Depicted is the receptor structure, and associated domains, of SR-AI, a representative of the SR-A group. Note that, in the case of SCARA4, a C-type lectin domain replaces the SRCR domain, while SCARA3 contains neither. CARD = caspase activation and recruitment domain; CTD = C-terminal domain; ECD = ectodomain; MDA5 = melanoma differentiation-associated gene 5; RIG-I = retinoic acid-inducible gene I; SRCR = scavenger receptor cysteine rich domain; TIR = Toll/IL-1 receptor domain; TLRs = Toll-like receptors; TM = transmembrane domain [2,8,9,10].

This review will discuss the cellular PRRs by family, including: TLRs (endosomal and surface), RLRs, CDSs and SR-As with their respective ligands, adaptor proteins, and downstream signaling pathways discussed afterwards. Figure 2 schematically demonstrates the key domains vital for PAMP recognition or downstream signaling activation for each PRR described within the present review and Table 1 summarizes the current knowledge of PRR identification in key fish species.

**Table 1:** Currently validated nucleic acid sensing pattern recognition receptors (PRRs) in model fish species. Eight model fish species are listed along the top row of the table; the PRR families and specific PRRs are listed along the left column. The validation criteria required the receptor to be amplified from tissue or a cell line at the transcript level at least, unless otherwise noted, in a peer-reviewed journal; the article containing this evidence is cited in the table. Sequences identified only through bioinformatic searches were not included. A cross bar indicates that the PRR has not yet been identified in that particular fish species. Abbreviations: Toll-like receptors (TLRs), RIG-I-like receptors (RLRs), Class A scavenger receptors (SRAs), Cytosolic DNA sensors (CDS).

Pattern Recognition Receptor		Atlantic Salmon *S. salar*	Grass Carp *C. idella*	Channel Catfish *I. punctatus*	Fugu *T. rubripes*	Japanese Flounder *P. olivaceus*	Large Yellow Croaker *L. crocea*	Rainbow Trout *O. mykiss*	Zebrafish *D. rerio*
TLRs	TLR3	✔^[11]^	✔^[19]^	✔^[28]^	✔^[33]^	✔^[34]^	✔^[42]^	✔^[48]^	✔^[53]^
	TLR7	✔^[12]^	✔^[20]^	✔^[29]^	✔^[33]^	✔^[35]^	✔^[43]^	✔^[49]^	✔^[53]^
	TLR8	✔^[13]^	✔^[21]^	✔^[30]^	✔^[33]^	✔^[36] **^	✔^[43]^	✔^[49]^	✔^[53]^
	TLR9	✔^[14]^	✔^[22]^	✔^[29]^	✔^[33]^	✔^[37]^	✔^[44]^	✔^[50]^	✔^[53]^
	TLR13	✔^[11]^		✔^[30] *^					
	TLR21	✔^[15]^	✔^[23]^	✔^[31]^	✔^[33]^	✔^[36] **^			✔^[53]^
	TLR22	✔^[16]^	✔^[24]^	✔^[29]^	✔^[33]^	✔^[38]^	✔^[45]^	✔^[16]^	✔^[53]^
RLRs	RIG-I	✔^[17]^	✔^[25]^	✔^[32]^					✔^[54]^
	MDA5	✔^[18]^	✔^[26]^	✔^[32]^		✔^[39]^	✔^[46]^	✔^[51]^	✔^[55]^
	LGP2		✔^[27]^	✔^[32]^		✔^[40]^		✔^[51]^	✔^[55]^
SRAs	SCARA3						✔^[47]^		
	SCARA4							✔^[52]^	✔^[56]^
	SCARA5						✔^[47]^		
	MARCO						✔^[47]^		✔^[57]^
CDS	DDX41					✔^[41]^			

* Identified through transcriptome shotgun analysis, however was not identified in tissue study [29]; ** Identified in genome through BAC analysis with probe analysis support.

### 2.1. Toll-Like Receptors (TLRs)

The toll-like receptors (TLRs) are a family of transmembrane proteins that consist of 19–25 tandem copies of the leucine-rich repeat (LRR) motif [58]. The LRR domain is capped on the N- and C- terminals by type-specific motifs [58]. TLRs can be divided into two broad categories: (i) those that recognize microbial lipids, sugars, and proteins, and (ii) those that recognize nucleic acid derivatives of viral or bacterial origin [59]. To date, 13 TLRs have been identified in mice, 10 TLRs in humans and 20 TLRs in fish including: TLR1, 2, 3, 4, 5M (membrane bound), 5S (soluble), 7, 8, 9, 13, 14, 18, 19, 20, 21, 22, 23, 24, 25, and 26 [60]. Some TLRs, such as TLR6 and TLR10, have been identified in mammals but do not appear to be present in fish. While these 20 TLRs have been identified in fish, the ligand specificities for many TLRs in fish remain unclear. Based on experimental data, the nucleic acid specific TLRs in fish appear to be: TLR3, which recognizes viral dsRNA intermediates, TLR7 and TLR8, which recognize ssRNA and TLR9, which recognizes unmethylated CpG DNA from viral or bacterial pathogens [2,61]. The ligand for TLR13 is thought to be 23S rRNA, but further research is required for clarification of this receptor’s ligand specificity [62]. The fish specific nucleic acid binding TLRs include: TLR21, which is found in chicken and fish, but not mammals, and is believed to play a similar role as TLR9 in recognizing unmethylated CpG DNA [63], and TLR22, which functions as a surface receptor for dsRNA [64]. What follows is a description of the nucleic acid-specific TLRs in fish, with particular attention to their mammalian counterparts in order to highlight the many similarities and differences between the two.

#### 2.1.1. Endosomal TLRs

In mammals, all nucleic acid-sensing TLRs are endosomal, with the exception of TLR3, which has been detected on the cell surface of some cell types [65,66,67], and are localized to the endoplasmic reticulum prior to PAMP stimulation, after which they move to an endosome for activation [68]. Non-mammalian PRRs, such as chicken TLR21, follow the same localization pattern [69] and TLR19 and 20 were found intracellular in salmon cells [15]. While it is often assumed that fish PRRs are located in the same compartments as their mammalian or avian counterparts, there is limited empirical verification of this assumption. Studies using chloroquine, a compound that blocks endosomal acidification, suggest that TLR3 and TLR9 are endosomal in fish models [49,70]. There is contradictory evidence seen with fish TLR7/8, as the effects of the TLR7/8 agonist R848 were inhibited by chloroquine treatment in Japanese flounder (*Paralichthys olivaceus*) [71], but not in rainbow trout (*Oncorhynchus mykiss*) [49]. The cellular location of other nucleic acid sensing TLRs has yet to be described in fish.

Endosomal TLRs can gain access to extracellular PAMPs, such as dsRNA, ssRNA, and DNA, when they are brought into the cell through scavenger receptor binding and endocytosis; this will be discussed more thoroughly in later sections of this review [72,73]. Autophagy is a process by which cells digest proteins or organelles from their cytosol, but also enables them to deliver cytosolic PAMPs to their cognate endosomal receptors [74]. Cytosolic PAMPs are sequestered into an autophagosome and are topologically inverted to bring the PAMPs to the same side of the membrane as the receptor portion of the endosomal TLRs [74].

##### 2.1.1.1. TLR3

TLR3 is a dsRNA sensor found in mammals and fish and is activated by viral dsRNA or synthetic dsRNA molecules, such as polyinosinic:polycytidylic acid (poly I:C). TLR3 activates the type I IFN and inflammatory pathways via the adaptor molecule toll-like receptor adaptor molecule 1 (TICAM-1), and subsequent activation of the transcription factors IRF3 and nuclear factor-kappa B (NF-κB) [75]. In fish, TLR3 recognizes virus sequence-derived dsRNA and poly I:C [64,76]. TLR3 sequences have been identified in many fish species, (Table 1). TLR3 transcripts have a broad tissue distribution in fish. For instance, in Atlantic salmon (*Salmo salar*), TLR3 is found in the head kidney, liver, heart, gill, muscle, gut, and spleen; in zebrafish TLR3 was found in the gill, spleen, kidney, heart, brain, and liver [11,77]. Poly I:C treatment or virus infection induces increased TLR3 expression in fish [42,76,78]. Overexpression of TLR3 in Japanese flounder, followed by poly I:C treatment, resulted in an increase in pro-inflammatory and IFN stimulated genes (ISGs) expression [34]. TLR3 knockdown in Japanese flounder resulted in a decreased poly I:C-induced immune response and antiviral activity [79]. Thus, TLR3 mediates antiviral activity in fish in response to dsRNA, similar to what is observed in mammals.

##### 2.1.1.2. TLR7/8

TLR7 and TLR8 both belong to the TLR7 family and have similar roles in the innate immune response. The ligand specificity of these receptors has some variation based on species; however, the ligand is typically ssRNA [2,80]. Mouse TLR7, as well as human TLR7, and -8, have been shown to respond to ssRNA [80,81]. Murine TLR8 does not respond to the human TLR7/8 agonist R848 or ssRNA and is therefore believed to be nonfunctional, suggesting there are differences in the receptors between species [80,82]. Mice deficient in (myeloid differentiation factor 88) Myd88 (a TLR adaptor molecule) or TLR7 no longer respond to R848 [82]. While human TLR7 and TLR8 both recognize similar ligands, TLR7-specific agonists induce IFN-α and IFN-regulated chemokines, and TLR8-specific agonists induce pro-inflammatory cytokines and chemokines [83].

TLR7 and TLR8 have been identified in fish and are present in many fish species (Table 1). TLR7 has a broad tissue distribution; Atlantic salmon TLR7 transcripts have been identified in the head kidney, liver, gut, and spleen; with strongest expression seen in the spleen and head kidney [11]. Grass carp TLR8 was found in all tissues tested, this included immune tissues, such as spleen and head kidney [84]. In yellow croaker, TLR7 and TLR8 were constitutively expressed in all tissues, with higher expression seen in immune tissues [43].

Currently there is no evidence for direct binding of TLR7 or TLR8 to their cognate ligands in fish models [59]. When TLR8 was knocked down in a grass carp cell line, down-regulation of IFN transcripts and no change in expression of MyD88, IRF7, or Mx1 were observed; however, TLR8 knockdowns were highly resistant to grass carp reovirus (GCRV) infection [84]. This may suggest TLR8 is a negative regulator of the antiviral response though more research is required to support this hypothesis [84]. Several single nucleotide polymorphisms (SNPs) were identified in grass carp TLR8 and two of the SNPs exhibited a correlation with resistance/susceptibility in grass carp to GCRV, which supports the role of TLR8 in antiviral immunity, however it does not shed light on any potential positive or negative regulations [21]. R848 is a specific TLR7/8 agonist, which, in Japanese flounder, caused inhibition of viral replication, enhanced cell proliferation, and reduced apoptosis [71]. The effects of R848 were blocked by treatment with chloroquine, which prevents endosomal acidification, suggesting that TLR7/8 in fish require endosomal maturation to activate [71]. Inhibition of MyD88 activation impaired the proliferation and anti-apoptosis effects of R848; when NF-κB was inactivated, these effects were significantly decreased. These results suggest that the immune response from R848 in Japanese flounder is likely due to TLR7/8 signaling, which is MyD88 and NF-κB dependent [71]. In grass carp kidney cells, TLR7 transcripts were induced by virus infection or poly I:C treatment, which is interesting as poly I:C is a dsRNA molecule [20]. Poly I:C also induced TLR7 and TLR8 expression in yellow croaker and grass carp [43,84]. In mammals, there is no evidence that poly I:C induces TLR7 or TLR8; however, poly I:C responses were not affected by TLR7 knockout in mice [80]. While TLR7 and TLR8 appear similar to their mammalian counterparts, further research is needed to understand if there are differences in pathway activation between the receptors, such as those seen in mammals, and to fully understand the role of TLR8 in the antiviral response.

##### 2.1.1.3. TLR9

TLR9 is a member of the TLR7 family that, in mammals, is required for recognition of unmethylated CpG motifs in synthetic oligodeoxynucleotides (ODNs), as well as bacterial and viral DNA [85]. Activated TLR9 signals through MyD88 and NF-κB to induce a pro-inflammatory response and expression of type I IFNs [86]. In murine models, the length of CpG-DNA is related to immunostimulatory potential, and in regards to ODNs, double-stranded ODNs are only weakly stimulatory compared to their single-stranded counterparts [87,88]. Macrophages have length-dependent mechanisms for the uptake of DNA and longer ODNs were taken up more efficiently [87].

TLR9 has been identified in many fish species (Table 1), and its expression profile in teleost species, such as zebrafish, Atlantic salmon and rainbow trout, is relatively broad irrespective of tissue type and developmental stage [60]. In unstimulated gilthead seabream (*Sparus aurata*), there were high levels of TLR9 transcript expression in gill, head kidney and spleen [89]. Injection of cobia (*Rachycentron canadum*) with ODNs induced high levels of TLR9 expression and pro-inflammatory cytokines in the spleen and liver [90]. In salmon, TLR9 transcript expression was upregulated in head kidney leukocytes after *in vitro* stimulation with CpG ODNs [14]. However, contrary to what was seen in cobia, when Atlantic salmon were treated *in vivo* with CpG ODNs, only a minor fold-change increase in TLR9 gene expression was observed in the spleen and head kidney [91]. TLR9 has been identified in zebrafish and upregulated in response to treatment with CpG ODNs [53,63].

In fish, the ligand specificity of TLR9 remains unclear [60]. In Atlantic salmon, plasmid DNA and synthetic unmethylated CpG ODNs induced an immune response, but GpC ODNs and methylated CpG motifs did not [92]. These results correspond with results in Japanese flounder cells where CpG motifs had a stimulatory effect, but GpC motifs did not [37]. In Atlantic salmon, a pull-down approach determined TLR9 could bind CpG ODNs; however, results demonstrated that TLR9 could bind both CpG and GpC ODNs similarly [60]. As in mammals, pH is important for TLR9 binding interactions in fish. In Atlantic salmon, TLR9 appeared to interact with synthetic ODNs through a pH-dependent, but CpG-independent mechanism [93]. Thus, the ability of fish cells to respond to CpG motifs requires further elucidation as contradictory evidence of TLR9’s binding abilities exist; the role of fish TLR9 in the antiviral response is even less well understood.

##### 2.1.1.4. TLR13

TLR13 is a recently identified endosomal PRR that is expressed in mice, but has not yet been identified in humans [62]. TLR13 is a PRR that recognizes bacterial 23S rRNA with a CGGAAAGACC motif, and is expressed in the spleen, dendritic cells, and macrophages [94]. In mice, TLR13 recognizes 13 residues within the V domain of 23S rRNA and appears to be unique among the nucleic acid sensors in that its ligand binding is sequence-specific [62]. Initial work suggests that TLR13 induces IL-1β through a MyD88-dependent pathway [62]. TLR13 does not however, exclusively sense bacterial PAMPs. In response to vesicular stomatitis virus (VSV) infection, TLR13 activated a MyD88-dependent pathway, resulting in the activation of NF-κB and type I IFN through IRF7, and cells without TLR13 are highly susceptible to VSV infection [95]. TLR13 has been identified in Atlantic salmon (Table 1) and channel catfish via cDNA library or transcriptome analysis; although TLR13 was not identified in catfish tissue samples as it was in Atlantic salmon tissues [29,30,96], specifically in the head kidney and spleen [11]. As this receptor has been recently identified, more studies will need to be completed to determine the ligand specificity in fish and the molecules involved in TLR13 signaling.

##### 2.1.1.5. TLR21

TLR21 is a non-mammalian PRR that binds CpG DNA and is found within the endosome in chickens [69]. TLR21 is present in a variety of fish species (Table 1). When TLR21 was knocked-down in chicken cells, ODN mediated responses were reduced [69,97]. Likewise, when chicken TLR21 was overexpressed in HEK-293 cells there was activation of NF-κB following CpG-ODN stimulation [69,97]. In fish, TLR21 is expressed mainly in organs related to the immune system, including the spleen [98]. The function of TLR21 is not yet fully understood. Studies in chickens suggest that TLR21 has a similar function to TLR9. Interestingly, chickens lack TLR9, while fish express both receptors [63,69]. In zebrafish, there is evidence that both TLR9 and TLR21 respond to CpG-ODN. Using the PRR ectodomains, it was determined that TLR9 had a broader sequence specificity range than TLR21, which had a preferred CpG motif [63]. TLR21 was identified in rock bream and was found to be expressed in immune tissues, and upregulated *in vivo* in response to rock bream iridovirus [99]. Atlantic salmon TLR21 was down-regulated in response to infectious salmon anemia virus infection [15]. TLR21 represents a uniquely non-mammalian PRR.

#### 2.1.2. Surface TLRs

Some TLRs, such as TLR3, are expressed on the cell surface in some cell types [66]. However, it is important to note that TLRs require maturation in an acidified endosome for ligand binding [100]. Thus, nucleic acid-binding TLRs are considered endosomal PRRs, as this is where their ligand binding capabilities exist. The exception to this rule appears to be the fish-specific receptor TLR22.

##### TLR22

TLR22 is a surface-expressed PRR involved in the recognition of dsRNA, and its expression is exclusive to teleost fish [64]. TLR22 belongs to the TLR11 family, however it appears to function similarly to TLR3, and signals through TICAM to induce IFN [64]. TLR22 is specific to fish and has been found in many species (Table 1). In turbot, TLR22 was constitutively expressed in a broad range of tissues, with higher transcript levels identified in kidney, head kidney, and spleen [101]. In gilthead seabream, poly I:C treatment did not lead to upregulation of TLR22 in acidophilic granulocytes, but did induce its expression in macrophages [102]. TLR22 expression was upregulated in the turbot gills, head kidney, spleen, and muscle following treatment with poly I:C, lipopolysaccharide (LPS, a bacterial PAMP), or turbot reddish body iridovirus infection [101]. Rohu (*Labeo rohita*) TLR22 was constitutively expressed in all embryonic developmental stages and in all tissues. Interestingly, poly I:C stimulation only resulted in significant TLR22 upregulation in the liver [103]. Fugu TLR22 expressed in human cells exhibited a dose-dependent increase in IFN-β activity following poly I:C treatment [64]. The length of the dsRNA molecule appears to affect TLR22-mediated antiviral responses, as IFN-β activity was greatest with dsRNA molecules of 1000 bp in length [64]. More data is needed to confirm the ligand specificity of TLR22 in fish species as its expression was induced by both bacterial and viral PAMPs (and by both nucleic acids and lipoglycans).

#### 2.1.3. Downstream Signaling: MyD88 and TICAM-1

Once the TLRs are activated, adaptor proteins are recruited to their cytosolic tails [77]. These adaptor proteins include MyD88 and TICAM-1 (also known as TIR-domain-containing adapter-inducing interferon-β, TRIF); simply put, MyD88 and TICAM-1 mediate the inflammatory and type I IFN responses within the cell. MyD88 sequences have been identified in fish species including: Atlantic salmon [13], grass carp [104], Japanese flounder [105], large yellow croaker [106], rainbow trout [107], and zebrafish [108]. MyD88-expression was induced in peripheral blood leukocytes of Japanese flounder following stimulation with poly I:C [105]. MyD88-binding to an activated PRR leads to activation of the mitogen-activated protein kinase (MAPK) signaling cascade, NF-κB activation and translocation followed by expression of pro-inflammatory cytokines [76]. Some of the important signaling proteins of the MyD88-activated signaling pathways include IRAK-4, a protein kinase, and TRAF6, an E3 ubiquitin protein ligase [76,109]. IRAK-4 and TRAF6 have been cloned in zebrafish. Overexpression of IRAK-4 or TRAF6 resulted in stimulation of NF-κB in zebrafish cells and TRAF6 transcript levels increased following infection with snakehead rhabdovirus [76]. When orange-spotted grouper IRAK-4 was over-expressed in human HEK-293 cells in conjunction with MyD88, there was significant impairment of NF-κB activity [110]. This suggests that IRAK-4 may have a different role in signal transduction in fish compared to its mammalian counterparts, possibly because grouper IRAK-4 lacks IRAK-2 interaction sites and has one altered autophosphorylation site [110]. In Indian major carp, MyD88 and TRAF6 were found in a broad range of tissues [111]. In addition to the NF-kB mediated inflammatory response, MyD88 has also been shown to interact with the type I IFN pathway associated transcription factors, interferon regulatory factor (IRF)-3 and -7. Interestingly, a synergistic effect on IFN promoter activity was observed between MyD88 and IRF-3 while MyD88 and IRF-7 associates resulted in attenuated promoter activity [112].

While the majority of TLRs signal through MyD88, TLR3 and TLR22 signal through TICAM-1. TICAM has been identified in multiple species including channel catfish [113], fugu [64], and zebrafish [114]. TICAM-1 in zebrafish localizes to the Golgi apparatus, this has not been observed in other fish or mammalian models [114]. TICAM-1 is able to activate the NF-κB response promoter in zebrafish [114] and activates the IRF3- and IRF7-mediated pathways [115]. Zebrafish TICAM-1 lacks N-terminal and C-terminal proline-rich domains found in the mammalian protein [114]. Fugu TLR3 and TLR22 induce type I IFN through interactions with TICAM-1; poly I:C stimulation causes TLR3 and TLR22 to recruit TICAM-1 [64]. While fish TICAM-1 does appear to have similarities to its mammalian counterpart such as its activation of specific signaling pathways and IFN production; however, there are structural differences between the two [116].

### 2.2. RIG-I-Like Receptors (RLRs)

The retinoic acid-inducible gene I (RIG-I)-like receptors (RLRs) are a family of DExD/H RNA helicases that sense PAMPs within the cytoplasm [115]. RIG-I and melanoma differentiation-associated protein 5 (MDA5), as well as Laboratory of Genetics and Physiology 2 (LGP2) are the three members of the family, and are involved in the recognition of dsRNA molecules. LGP2 lacks a functional caspase-recruitment domain (CARD), and therefore may not be directly involved in downstream signaling [117].

#### 2.2.1. RIG-I and MDA5

RIG-I and MDA5 are both dsRNA activated receptors; RIG-I also recognizes ssRNA with 5' triphosphorylated or 5' disphosphorylated ends and polyuridine sequences [3,118,119]. RIG-I exists in an inactive state in a resting cell, folded on itself. Upon ligand binding to the helicase and C-terminal domain (CTD), the protein opens to expose its CARD domains, which bind its adaptor protein, IFN promoter stimulator (IPS-1). Activated IPS-1 initiates IFN-mediated signaling pathways via IRF3/7 [2]. RLRs can also play a role in the pro-inflammatory pathway; MDA5 and RIG-I are critical for inducing pro-inflammatory cytokines in addition to type I IFN production [120,121].

A variety of fish have RIG-I and MDA5 (Table 1). Zebrafish express RIG-Ib, a typical RIG-I, and RIG-Ia, an insertion variant [122]. RIG-I knockdown in the zebrafish cell line, ZF4, resulted in lower activity of group II type I IFNs and down-regulation of the inflammatory response in nervous necrosis virus (NNV)-infected cells [123]. In ZF4 cells, RIG-Ia and RIG-Ib were upregulated in response to spring viremia of carp virus (SVCV) infection [122]. Overexpression of zebrafish RIG-I in epithelioma papulosum cyprini (EPC) cells resulted in significant type I IFN promoter activity and enhanced Mx and IRF7 expression [122]. EPC cells expressing RIG-Ib produced lower virus yields compared to RIG-Ia or control cells, suggesting the insert variant is more effective at inducing an antiviral state even though RIG-Ib was more effective at inducing IFN promoter activity [122]. Zebrafish RIG-Ia and RIG-Ib represent the first identified RIG-I variants in vertebrates [122]. RIG-I and MDA5 transcripts were upregulated during snakehead fish vesiculovirus (SFV) infection in ZF4 cells [124]. Activation of crucian carp viperin (an ISG) promoter by intracellular poly I:C is mediated by an RLR-triggered IFN pathway [125], suggesting the RIG-I pathway signals similarly in fish as in mammals.

MDA5 is homologous to RIG-I and exhibits the same domain structure; however, MDA5 preferentially binds longer lengths of dsRNA (>1000 bp) and RIG-I preferentially binds shorter dsRNA (<1000 bp) [126]. It is not fully understood why there is a difference in their binding of different lengths, however one hypothesis suggests that long poly I:C molecules are able to bind to RIG-I, but they induce a conformation change that makes RIG-I unable to participate in downstream signaling [127]. In mammals, MDA5 induces IFN and ISG transcript expression via the IPS-1 and IRF3/7 pathway, similar to that of RIG-I [128,129]. Similar pathways appear to be present in fish as well. In Japanese flounder, *in vitro* knockdown of MDA5 in flounder gill cells and *in vivo* knockdown followed by spleen analysis exhibited reduced poly I:C-mediated immune responses and antiviral activity [79]. MDA5 from green chromide (*Etroplus suratensis*) was ubiquitously expressed in unstimulated fish; the highest expression was seen in the muscle; however, there was also high expression in the spleen, head kidney, and hindgut [130]. Poly I:C injection resulted in upregulation of MDA5 in tissues, there was significant upregulation in the spleen, liver, kidney, intestine, heart and gill over 48 h [130]. Two splice variants of MDA5 are found in zebrafish, MDA5a and MDA5b, and both transcripts were upregulated in fish cell lines following infection with SVCV [131]. Overexpression of MDA5a and MDA5b in EPC cells resulted in significant induction of type I IFN-promoter activity and enabled protection of transfected EPC cells against virus infection [131]. When MDA5b was co-transfected with MDA5a or IPS-1 there was higher IFN-promoter activity compared to the control and MDA5b alone [131]. Although fish have two variants of RIG-I and MDA5, they appear to participate in the innate immune pathways in a similar manner to their mammalian homologs.

#### 2.2.2. LGP2

LGP2 is the third member of the RLR family. LGP2 plays an interesting role in the dsRNA-mediated pathway, as it interacts with dsRNA but has no N-terminal CARD domain, suggesting that it is not directly involved with downstream signaling [132]. Initial evidence suggested that LGP2 was a negative regulator of RIG-I and MDA5, but more recent data shows that LGP2 facilitates IFN production in response to RNA viruses [132]. In mammals, LGP2 assists MDA5-RNA interactions [133]. LGP2^−/−^ mice exhibited resistance to VSV, but displayed defective type I IFN production when infected with encephalomyocarditis virus (ECMV) [134]. Overall, the role of LGP2 in the innate antiviral response is still unclear in mammals, as well as in fish, as it has both inhibitory and stimulatory effects depending on the stimulus.

LGP2 has also been identified in many different fish (Table 1). Following viral challenge, LGP2 enhanced MDA5 and IPS-1 expression, and the antiviral immune response, though it impaired the expression of RIG-I in grass carp kidney cells [135]. Overexpression of LGP2 inhibited grass carp reovirus (GCR) replication and protected the cells from viral infection. Following poly I:C stimulation, LGP2 induced RIG-I transcripts and inhibited MDA5 transcripts [135]. In the common carp, LGP2 showed widespread expression and transcript levels increased after infection with koi herpes virus (KHV) or poly I:C treatment [136]. Two isoforms of LGP2 have been identified in zebrafish and rainbow trout [51,124]. In rainbow trout, overexpression of only one variant resulted in enhanced protection against viral infection [51]. SFV infection in ZF4 cells upregulated the isoform LGP2a protein and transcripts, but did not affect LGP2b expression [124]. While LGP2 appears to play a role in the RLR-mediated immune pathway in fish, its role in the pathway remains to be elucidated.

#### 2.2.3. Downstream Signaling: IPS-1

In mammals, MDA5 and RIG-I both induce IFN production through the mitochondrial adaptor IPS-1 (also known as MAVS, VISA or CARDIF) [3]. This pathway appears to be conserved in fish as well. The adaptor protein IPS-1 was cloned from green chromide, constitutively expressed in many tissues as well as being upregulated in response to poly I:C injection [130]. Atlantic salmon IPS-1 was shown to mediate the activation of both the NF-κB and IFN-α1 promoters [137]. Overexpression of IPS-1 in Japanese flounder resulted in a protective antiviral state; overexpression specifically led to IRF3 and ISGs expression [138]. Stimulation with poly I:C or virus infection resulted in enhanced IPS-1 expression in grass carp [104]. This was not seen in crucian carp, where IPS-1 was found to be constitutively expressed and was not induced by poly I:C or IFN; however, blocking IPS-1 inhibited the response to poly I:C or IFN [139]. Taken together, these results suggest that in crucian carp IPS-1 mediates the IFN-mediated response downstream of poly I:C and RIG-I and upstream of IRF3/7, similar to what is observed in mammals [139]. IPS-1 overexpression in EPC cells resulted in increased expression of IFN and ISGs, downstream of RIG-I [139]. Two IPS-1 alleles are found in zebrafish, and both variants activate the IFN promoter [55]. There is evidence of a cooperative effect between IPS-1 and RIG-I or MDA5 within zebrafish [55]. The presence of IPS-1 in many fish models and its similarities to mammalian IPS-1 suggest a conserved pathway of IFN expression.

#### 2.2.4. Downstream Signaling: IRF3/7

The IFN regulatory factors (IRFs) are a group of transcription factors possessing an N-terminal helix-turn-helix DNA binding motif [140,141]. In mammals, the homologs IRF3 and IRF7 are regulators of type I IFN expression [141]. Members of both the TLR and RLR families signal through IRF7- or IRF3-mediated pathways in fish [115]. IRF3 and IRF7 have been identified in rainbow trout and contain a DNA-binding domain; both IRF3 and IRF7 were upregulated in response to poly I:C as well as recombinant type I and type II IFN [128]. Crucian carp (*Carassius carassius*) IFN treatment leads to IRF3 phosphorylation and nuclear translocation. Additionally, fish IRF3 functions as an ISG, in addition to an IFN transcription factor, which is not the case for mammalian IRF3 [142]. Overexpression of crucian carp IRF3 activates production of IFN, which in turn triggers ISG transcription; transfection of cells with a carp dominant negative IRF3 abolishes poly I:C induction of ISGs [142].

### 2.3. Cytosolic DNA Sensors (CDSs)

While the immunostimulatory effects of DNA have long been recognized, it is only in the past several years that the role of DNA sensing in innate immunity has come to light. In mammals, the recognition of endosomal DNA is accomplished by TLR9, whereas recognition of cytosolic DNA appears to involve several intracellular receptors, collectively referred to as cytosolic DNA sensors (CDSs). These receptors include: DNA-dependent activator of IFN-regulatory factors (DAI) [143], absent in melanoma 2 (AIM2) [144,145,146], RIG-I via RNA polymerase III (RNA pol III) [7,147], leucine-rich repeat (in Flightless I) interacting protein-1 (Lrrfip1) [148], DExD/H-box helicases DDX41, DHX9, and DHX36 [149,150], cyclic GMP-AMP synthetase (cGAS) [151], and IFI16 (also known as p204) [6,152,153]. Some CDSs mediate an IFN-dependent pathway. DAI recognizes cytosolic dsDNA directly and induces type I IFN expression [143,153]. RNA pol III is able to transcribe viral DNA into a 5' triphosphate RNA intermediate, which is capable of inducing type I IFNs via RIG-I [7,147]. DExD/H-box helicases, such as DHX9 and DHX36, bind directly to CpG-DNA and, like TLR9, interact with MyD88 in a TLR9-independent manner [148]. Using a human monocytic cell line with limited basal IFI16 expression (THP-1), Zhang *et al.* (2011) [149] found constitutively expressed DDX41 to be the initial sensor of cytoplasmic DNA, which subsequently activates IFN and IFI16 to amplify the innate antiviral response. cGAS, a c-GAMP synthase, is capable of binding cytosolic DNA and inducing IFN [150]. Lrrfip1 is a unique CDS in that it recognizes both dsRNA and dsDNA and induces IFN expression without directly regulating the IFN transcription factors, instead, using Lrrfip1-targeting siRNA [148]. Other CDSs, described as the AIM2-like receptors (ALRs), mediate a pro-inflammatory response. These include AIM2 and IFI16, which induce maturation of IL-1b through inflammasome formation (discussed below) [151,154].

Of all of the aforementioned CDSs, only DDX41 has been identified in teleost fish [41]. A recent study by Quynh *et al.* 2015 [41] identified DDX41 as a CDS in Japanese flounder (*Paralichthys olivaceus*). The DDX41-encoding gene was identified as an ancestral cytoplasmic viral DNA sensor that exhibits antiviral function similar to that in mammals. Japanese flounder DDX41 is expressed in a variety of tissues (heart, kidney, liver, stomach), and is induced by ranavirus (a DNA virus) infection. Moreover, Quynh *et al.* (2015) also demonstrated that the Japanese flounder DDX41 functions primarily in monocytes [41], consistent with previous findings in mammals [149]. Lastly, Japanese flounder DDX41 was shown to induce the expression of antiviral (IFN, Mx) and inflammatory (IL-6, IL-1b) cytokines following stimulation with C-di-GMP (dinucleotides) [41]. Taken together, these results suggest that the Japanese flounder CDS, DDX41, induces IFN-mediated antiviral and inflammatory responses, similar to mammals. To the authors’ knowledge, no other CDSs have been identified in teleost fish. Thus, whether these newly described DNA sensors are present in the fish cytosol, and function similarly to their mammalian counterparts, is the subject of ongoing research.

#### Downstream Signaling: STING

For the numerous CDSs that have been described, signaling is mediated by a number of adaptor molecules, which transmit signals to NF-kB and members of the IRF family to induce inflammatory and antiviral responses, respectively. DDX41 induces type I IFN production by signaling through the endoplasmic reticulum (ER)-associated stimulator of IFN genes (STING) protein (also called MITA, mediator of IRF3 activation; ERID, endoplasmic reticulum IFN stimulator; and MPYS, N-terminal methionine-proline-tyrosine-serine protein) [155]. STING is regarded as the key adaptor protein for cytosolic DNA sensing (reviewed in [156]). Previous studies have shown that STING overexpression enhances type I IFN and ISG expression, whereas STING deficiency inhibits the innate antiviral response, causing increased susceptibility to both RNA and DNA viruses [157,158,159,160,161]. Moreover, STING has been shown to directly bind self and foreign DNA to stimulate downstream signaling characterized by the activation of antiviral and inflammatory genes, including type I IFN [162]. In this sense, STING may itself be considered a novel CDS. While CDSs themselves have not been well characterized in teleost fish, STING orthologs have been identified in goldfish (*Carassius auratus*), zebrafish, and fathead minnow (*Pimephales promelas*), and found to function analogously to mammalian STING in the induction of IFN and ISGs and protection against RNA and DNA viruses [155,163]. As well, both groups reported that STING was a key component of the RIG-I pathway of cytosolic RNA recognition in these fish. Downstream of their respective adaptor proteins, the DNA sensing pathways converge on the activation of IKK kinases, which go on to phosphorylate and activate IRFs (reviewed in [164]). STING has been shown to interact with the IKK kinase, TANK-binding kinase 1 (TBK1), which phosphorylates and activates IRF3 to induce IFN. As such, TBK1 is recognized as the central kinase for DNA recognition. With respect to teleost fish, TBK1 transcripts have been identified in zebrafish [165], crucian carp [163], Atlantic cod [166], and common carp [167]. It was shown that crucian carp TBK1 enhances type I IFN promoter activity in an IRF3- and IRF7-dependent manner [163]. This evidence suggests that fish possess a functional conserved STING-TBK1-IRF3-IFN pathway, capable of inducing type I IFNs in response to cytosolic nucleic acids.

### 2.4. Class A Scavenger Receptors (SRAs)

Scavenger receptors are cell surface receptors that were originally defined by their ability to bind and internalize modified low-density lipoproteins (mLDLs) [168], which play a major role in the development of atherosclerosis. For this reason, research has largely focused on their involvement in the development of vascular disease. Scavenger receptors are now recognized as a diverse group of PRRs that sense a variety of polyanionic ligands other than mLDLs, including dsRNA [169]. SR-As are type II membrane glycoproteins and consist of SR-AI/II/III, MARCO, SCARA3, SCARA4, and SCARA5. All members contain cytoplasmic, transmembrane, α-helical, and collagenous domains, though they differ in the lengths of their α-helical and collagenous domains, and in the composition of their C-terminal domains [8]. SR-AI, MARCO and SCARA5 also possess a terminal Scavenger Receptor Cysteine Rich (SRCR) domain, SCARA4 possesses a C-type lectin domain and SCARA3 terminates at the collagenous domain [8]. All 5 SR-A members form homotrimers, which are stabilized by α-helical coiled-coil motifs and by their collagenous regions [170]. In SR-AI, the collagenous region mediates ligand binding and therefore pathogen recognition [171], whereas the SRCR domain mediates this function in MARCO [172]. With the C-type lectin domain of the collectins [173] and the leucine-rich repeat of the TLRs [174], the SRCR domain is one of the most ancient pattern recognition domains associated with innate immunity. Although it is found in many proteins and highly conserved across various deuterosome species [175], no full SR-As have been identified in non-vertebrate genomes, indicating that the modern SR-A structure arose after the divergence of vertebrates from other species [8].

The role of SR-As in antiviral innate immunity is highlighted by their ability to bind the synthetic dsRNA poly I:C [176] and DNA [73]. SR-As have been shown to cooperate with TLRs to initiate cytokine secretion after nucleic acid recognition. MARCO also cooperates with TLRs as it has been shown to deliver CpG-DNA to endosomal TLR9 [73]. SR-As have been proposed to bind and internalize extracellular dsRNA through clathrin-mediated endocytosis [169]. Once within the endosome, dsRNA can be detected by TLR3, and by escaping the endosome via an unknown mechanism, can also be detected by RIG-I and MDA-5, which initiate TICAM- and IPS-1-dependent antiviral responses, respectively.

More research is required to elucidate SR-A function, specifically in nucleic acid binding and signaling in mammals. Even less is known in fish, though research to date does indicate that fish SR-A sequence, structure, and function share many similarities with mammalian SR-As [52]. Several SR-A sequences and homologs have been identified in fish, but more functional studies are needed to characterize their expression and function in different fish cells and tissues. *In silico* analysis has shown that SCARA5 is conserved in fish, SCARA3 is conserved specifically in Ostariophysian and Salmonidae fish species and SCARA4 is conserved in these genomes as well as in the Acanthopterygii fishes [8]. MARCO, SCARA3, and SCARA5 have been identified in large yellow croaker and their expression was upregulated *in vivo* following bacterial infection [47]. A SCARA4 homolog has been cloned in zebrafish, in which it binds both mLDLs and bacteria, and is involved in vasculogenesis [56]; a SCARA4 fragment has also been identified in rainbow trout [52,177]. A SCARA5 homolog has been cloned in puffer fish, and is able to bind LPS and negatively affect the pro-inflammatory response [178]. To date, no SR-AI/II/III sequences have been identified in fish [8]. Functionally, the same competitive ligands used to define SR-A function in mammals have been used to define function in fish, such as fucoidan, DxSO_4_ and formaldehyde-treated albumin (FSA) [52]. Based on functional studies, SR-As have been shown to bind ligands in Atlantic cod scavenger endothelial cells [179], catfish nonspecific cytotoxic cells [180], and rainbow trout kidney macrophages [181].

#### Downstream Signaling

While current research proposes that SR-As function predominantly as carriers to deliver dsRNA to TLR3, RIG-I and MDA-5, there is a possibility that SR-As are capable of initiating an antiviral response independent of TLRs and RLRs [169]. It has been shown that SR-A ligand binding causes tyrosine phosphorylation of phospholipase C-γ1 (PLC-γ1) and phosphatidylinositol 3-kinase (PI 3-kinase) and activates signaling pathways involving protein kinase C (PKC), heterotrimeric G_(i/o)_ proteins, mitogen-activated protein kinases (MAPKs), caspases and cytokine secretion [182,183,184,185]. However, fucoidan and lipoteichoic acid-mediated signaling pathways still occur in macrophages from mice lacking SR-A [186], suggesting that SR-As’ major function is to control ligand uptake and degradation rather than intracellular signaling [187]. Currently there is no evidence for SR-A signaling mechanisms in fish.

### 2.5. The Type I Interferon Response

Type I IFNs are autocrine and paracrine cytokines that inhibit viral replication and infection by inducing an “antiviral state” through the production of IFN-stimulated genes (ISGs) [188]. In mammals, the key antiviral IFN subtypes are IFN-α and IFN-β. In teleost fish, type I IFNs are referred to as group I and group II type I IFNs, depending on their cysteine composition [189]. Secreted IFNs signal through type I IFN receptors (IFNAR); these receptors are ubiquitously expressed cell surface receptors and, in mammals are heterodimeric, comprised of two subunits, IFNAR1 and IFNAR2 [188,190,191]. In mammals, IFN bound to IFNAR activates the Janus tyrosine kinases JAK1 and TYK2, which phosphorylate transcription factors, signal transducers and activators of transcription (STAT)1 and STAT2 [188]. The activated STATs form a heterodimer and recruit IFN-regulatory factor 9 (IRF9) to produce a STAT1-STAT2-IRF9 complex called ISGF3 [188,190]. This complex translocates into the nucleus and activates ISGs through binding to the IFN-sensitive response element (ISRE) within the promoter region of various ISGs [188].

dsRNA is a potent inducer of type I IFN in fish. Rohu type I IFN is sensitive to poly I:C induction [192]. When Japanese flounder cells, overexpressing MDA5, RIG-I, or IRF3, were treated with poly I:C there was an increase in type I IFN production [193]. Rock bream IFN was found to be induced by poly I:C or an iridovirus infection [194]. In grass carp, IRF7 was shown to bind to the promoter sequence of grass carp IFN and IRF7 acted as a positive regulator for the transcription of IFN1, supporting evidence that IRF7 regulates IFN production in fish; grass carp IFN was also upregulated by poly I:C treatment [195]. In addition to exogenous PRR ligand treatments, virus infections also induce IFNs in fish, as observed in mammals. Viruses have many evasion strategies to block IFN production to inhibit the innate immune response from being mounted against them. For example, SVCV induced high levels of type I IFN expression, while the Cyprinid herpesvirus 3 (CyHV-3) infections did not induce any response in the common carp brain (CCB) cells [196]. This suggests that CyHV-3 has an effective mechanism for blocking IFN production in these cells. Activation of type I IFN by poly I:C in CCB cells during CyHV-3 infection resulted in reduction of virus titer and spread of virus; so while the virus may have anti-IFN functions, it was still susceptible to the antiviral state induced by IFN [196].

Type I IFN stimulates the production of a series of genes referred to as ISGs. ISGs are a diverse group of proteins that block virus infection and replication through a varied set of complementary strategies [188]. Many fish ISGs are homologous to mammalian ISGs and display similar antiviral activities, these include Mxs and ISG15. Mx proteins are dynamin-related members of a GTPase family that have been identified in a broad variety of species, including mammals and teleost fish [197]. In mammals, Mx proteins inhibit virus replication through interaction with nucelocapsid-type structures and exhibit significant antiviral activity against many different viruses including VSV and the influenza virus [197,198,199,200]. Mx1 protein in Atlantic salmon inhibited the replication of infectious pancreatic necrosis virus (IPNV) and Mx in Japanese flounder inhibited fish rhabdoviruses [201,202]. ISG15 is an ubiquitin-like protein and attaches to target proteins through a C-terminal Gly-Gly motif [203]. ISG15 has been shown to have antiviral activity in orange-spotted grouper [204], zebrafish [205], and crucian carp [206]. GIGs (Grass carp hemorrhagic virus-induced genes) are fish specific ISGs originally identified in crucian carp [205]. GIGs have since been identified in zebrafish [207] and grass carp [208]. GIGs are still relatively understudied, compared to other ISG families, but represent an interesting difference between mammalian and fish immune systems.

### 2.6. The Inflammatory Response

In addition to the type I IFN response, viral nucleic acid PRRs induce an inflammatory response by a two steps process: (1) increasing expression of IL-1 and (2) triggering inflammasome formation to cleave IL-1 into its active forms, IL-1α and IL-1β. Activation of the transcription factor NF-κB by PRR-mediated signaling pathways induces IL-1 gene expression [209]. A second signal, mediated by PRRs such as AIM2 binding of dsDNA directly, triggers formation of an inflammasome and subsequent IL-1 cleavage [144,145]. In mammalian cells, the inflammasome is required for the activation of inflammatory caspases, namely caspase-1 (also called interleukin converting enzyme, ICE), which mediates processing of IL-1, a necessary prerequisite for their secretion and function [210].

While many of the features of the inflammasome and IL-1 biology have been characterized in mammals, much remains to be discovered regarding these mechanisms in fish. In mammals, there are multiple expansions of the IL-1 gene family including IL-1α, IL-1β, IL-1 receptor antagonist (IL-1RA) and at least six others [211]; however to date, only two IL-1 gene family members have fish homologues: IL-1β and its relative IL-18 [212], while fish may express a novel IL-1 receptor antagonist, nIL-1F [213]. Owing to the presence of an IL-1 family signature [214], as well as overall greater homology with IL-1β, IL-1 genes in fish are typically regarded as IL-1b orthologs. IL-1β has been identified in a host of teleost fish species including cyprinids [215], salmonids [216,217], perciformes [218,219,220,221,222], gadiformes [223], and anguilliformes [224]. A few studies have even indicated the presence of multiple IL-1β genes in rainbow trout [225], common carp [226], salmon [227], and catfish [228]. With respect to processing, evidence suggests that, like mammals, fish IL-1β is synthesized as an inactive pro-peptide that is subsequently cleaved for bioactivity [229,230]. However, the mechanism of IL-1β processing and maturation in fish is currently under debate. As mentioned, mammalian proIL-1β (IL-1) is cleaved by caspase-1 as part of an inflammasome complex to yield mature IL-β. However, with minor exceptions, all nonmammalian IL-1 genes described thus far lack the conserved mammalian caspase-1 cleavage site [214].

With respect to function, it appears that IL-1β behaves the same in teleost and mammalian immunity. As seen in mammals, fish IL-1 is upregulated in response to infection and various pro-inflammatory stimuli [214,231,232]. As well, fish IL-1β has an immunostimulatory effect similar to that of mammals [233,234,235]. It appears that, like mammals, fish IL-1 requires proteolytic cleavage for maturation into a biologically activated form [229,230,236,237,238].

## 3. Future Directions

While a lot has been elucidated regarding nucleic acid sensing in fish, much still remains to be understood. Many studies rely heavily on observing PRR induction, or measuring expression levels, following stimulation with candidate ligands. However, functional studies would help to further elucidate the complex interactions and highlight any differences that may exist between fish and their mammalian counterparts. The presence of contradictory reports regarding ligand specificity between fish species necessitates further investigation. For instance, the ability of fish cells to respond to CpG motifs is ambiguous, although this may partially be due to a poor understanding of TLR9 biology in general [60]. Also, novel teleost PRRs require further investigation as they are newly described, and thus poorly understood. Specifically, the ligand specificity and signaling mechanism for TLR13, TLR21, and TLR22 is poorly characterized in fish and requires further investigation in order to unravel the mysteries behind these novel PRRs. As outlined in Table 1, further work is required to identify PRRs from every nucleic acid sensing PRR family in fish; however, it is clear that CDSs are dramatically under studied in fish compared to mammals. Clearly fish are able to sense DNA and mount an antiviral response; however, what sensors are involved in these pathways need to be identified. Lastly, given that nucleic acid sensing uses a suite of PRRs with multiple family members, tissue and cell type specific expression needs to be investigated to fully understand how different cell types work together to sense and mount an antiviral response. While much progress has been made with respect to teleost nucleic acid sensing, much remains to be discovered in order to fully understand the antiviral response in the economically important animals.

## 4. Conclusions

The ability of fish cells to recognize and respond to nucleic acids from a variety of pathogens has been known for some time. Representative members of each nucleic acid PRR family, TLRs, RLRs, SR-As, and CDSs, have been described in fish, and have been shown to function similarly to their mammalian counterparts (Figure 1). This indicates an evolutionarily conserved system, demonstrating their importance in innate immunity. All of the TLRs, RLRs, and SR-As (with the exception of SR-AI/II/III) identified in mammals have been described in fish; however, only one CDS family member has been identified in fish. Fish also possess unique nucleic acid-sensing PRRs, such as TLR21 and TLR22, which are not expressed in mammals. While many of the downstream responses, including type I IFN induction and inflammatory cytokine production and release, parallel those seen in mammals, ligand binding specificities appear to vary. This may reflect differences in aquatic *vs.* terrestrial pathogens [64] or changes that have occurred during vertebrate evolution. Regardless, further research is required to elucidate the exact ligands for each of these fish PRRs. Despite differences between teleost and mammalian nucleic acid sensing, there is much resemblance, indicating an evolutionarily conserved system that is essential for pathogen detection and clearance.

## References

[1] Nellimarla S., Mossman K.L. (2014). Extracellular dsRNA: Its function and mechanism of cellular uptake. J. Interferon Cytokine Res..

[2] Jensen S., Thomsen A.R. (2012). Sensing of RNA viruses: A review of innate immune receptors involved in recognizing RNA virus invasion. J. Virol..

[3] Loo Y.M., Gale M. (2011). Immune signaling by RIG-I-like receptors. Immunity.

[4] Jacobs B.L., Langland J.O. (1996). When two strands are better than one: The mediators and modulators of the cellular responses to double-stranded RNA. Virology.

[5] DeWitte-Orr S.J., Mossman K.L., Mossman K. (2011). The antiviral effects of extracellular dsRNA. Viruses and Interferon: Current Research.

[6] Horan K.A., Hansen K., Jakobsen M.R., Holm C.K., Søby S., Unterholzner L., Thompson M., West J.A., Iversen M.B., Rasmussen S.B. (2013). Proteasomal degradation of herpes simplex virus capsids in macrophages releases DNA to the cytosol for recognition by DNA sensors. J. Immunol..

[7] Chiu Y.-H., MacMillan J.B., Chen Z.J. (2009). RNA polymerase III detects cytosolic DNA and induces type I interferons through the RIG-I pathway. Cell.

[8] Whelan F.J., Meehan C.J., Golding G.B., McConkey B.J., Bowdish D.M. (2012). The evolution of the class A scavenger receptors. BMC Evol. Biol..

[9] Botos I., Segal D.M., Davies D.R. (2011). The structural biology of Toll-like receptors. Structure.

[10] Bowie A.G. (2012). Innate sensing of bacterial cyclic dinucleotides: More than just STING. Nat. Immunol..

[11] Arnemo M., Kavaliauskis A., Gjøen T. (2014). Effects of TLR agonists and viral infection on cytokine and TLR expression in atlantic salmon (*Salmo salar*). Dev. Comp. Immunol..

[12] Lee P., Zou J., Holland J., Martin S., Kanellos T., Secombes C. (2013). Identification and characterization of TLR7, TLR8a2, TLR8b1 and TLR8b2 genes in Atlantic salmon (*Salmo salar*). Dev. Comp. Immunol..

[13] Skjaeveland I., Iliev D.B., Strandskog G., Jørgensen J.B. (2009). Identification and characterization of TLR8 and MyD88 homologs in Atlantic salmon (*Salmo salar*). Dev. Comp. Immunol..

[14] Skjaeveland I., Iliev D.B., Zou J., Jørgensen T., Jørgensen J.B. (2008). A TLR9 homolog that is up-regulated by IFN-γ in Atlantic salmon (*Salmo salar*). Dev. Comp. Immunol..

[15] Lee P., Zou J., Holland J., Martin S., Collet B., Kanellos T., Secombes C. (2014). Identification and characterisation of TLR18–21 genes in Atlantic salmon (*Salmo salar*). Fish Shellfish Immunol..

[16] Rebl A., Siegl E., Köllner B., Fischer U., Seyfert H.-M. (2007). Characterization of twin toll-like receptors from rainbow trout (*Oncorhynchus mykiss*): Evolutionary relationship and induced expression by *Aeromonas salmonicida salmonicida*. Dev. Comp. Immunol..

[17] Biacchesi S., LeBerre M., Lamoureux A., Louise Y., Lauret E., Boudinot P., Bremont M. (2009). Mitochondrial antiviral signaling protein plays a major role in induction of the fish innate immune response against RNA and DNA viruses. J. Virol..

[18] Sun B., Robertsen B., Wang Z., Liu B. (2009). Identification of an Atlantic salmon IFN multigene cluster encoding three IFN subtypes with very different expression properties. Dev. Comp. Immunol..

[19] Su J., Zhang R., Dong J., Yang C. (2011). Evaluation of internal control genes for qRT-PCR normalization in tissues and cell culture for antiviral studies of grass carp (*Ctenopharyngodon idella*). Fish Shellfish Immunol..

[20] Yang C., Su J., Zhang R., Peng L., Li Q. (2012). Identification and expression profiles of grass carp *Ctenopharyngodon idella* TLR7 in responses to double-stranded RNA and virus infection. J. Fish Biol..

[21] Su J., Su J., Shang X., Wan Q., Chen X., Rao Y. (2015). SNP detection of TLR gene, association study with susceptibility/resistance to GCRV and regulation on mRNA expression in grass carp, *Ctenopharyngodon idella*. Fish Shellfish Immunol..

[22] Yang C.-R., Su J.-G., Peng L.-M., Dong J. (2011). Cloning and characterization of grass carp (*Ctenopharyngodon idella*) toll-like receptor 9. J. Fish. China.

[23] Wang W., Shen Y., Pandit N.P., Li J. (2013). Molecular cloning, characterization and immunological response analysis of toll-like receptor 21 (TLR21) gene in grass carp, *Ctenopharyngodon idella*. Dev. Comp. Immunol..

[24] Lv J., Huang R., Li H., Luo D., Liao L., Zhu Z., Wang Y. (2012). Cloning and characterization of the grass carp (*Ctenopharyngodon idella*) toll-like receptor 22 gene, a fish-specific gene. Fish Shellfish Immunol..

[25] Yang C., Su J., Huang T., Zhang R., Peng L. (2011). Identification of a retinoic acid-inducible gene I from grass carp (*Ctenopharyngodon idella*) and expression analysis *in vivo* and *in vitro*. Fish Shellfish Immunol..

[26] Su J., Huang T., Dong J., Heng J., Zhang R., Peng L. (2010). Molecular cloning and immune responsive expression of MDA5 gene, a pivotal member of the RLR gene family from grass carp *Ctenopharyngodon idella*. Fish Shellfish Immunol..

[27] Huang T., Su J., Heng J., Dong J., Zhang R., Zhu H. (2010). Identification and expression profiling analysis of grass carp *Ctenopharyngodon idella* LGP2 cDNA. Fish Shellfish Immunol..

[28] Bilodeau A.L., Waldbieser G.C. (2005). Activation of TLR3 and TLR5 in channel catfish exposed to virulent *Edwardsiella ictaluri*. Dev. Comp. Immunol..

[29] Quiniou S.M., Boudinot P., Bengtén E. (2013). Comprehensive survey and genomic characterization of toll-like receptors (TLRs) in channel catfish, *Ictalurus punctatus*: Identification of novel fish TLRs. Immunogenetics.

[30] Liu S., Zhang Y., Zhou Z., Waldbieser G., Sun F., Lu J., Zhang J., Jiang Y., Zhang H., Wang X. (2012). Efficient assembly and annotation of the transcriptome of catfish by RNA-seq analysis of a doubled haploid homozygote. BMC Genomics.

[31] Baoprasertkul P., Xu P., Peatman E., Kucuktas H., Liu Z. (2007). Divergent toll-like receptors in catfish (*Ictalurus punctatus*): TLR5S, TLR20, TLR21. Fish Shellfish Immunol..

[32] Rajendran K., Zhang J., Liu S., Peatman E., Kucuktas H., Wang X., Liu H., Wood T., Terhune J., Liu Z. (2012). Pathogen recognition receptors in channel catfish: II. Identification, phylogeny and expression of retinoic acid-inducible gene I (RIG-I)-like receptors (RLRs). Dev. Comp. Immunol..

[33] Oshiumi H., Tsujita T., Shida K., Matsumoto M., Ikeo K., Seya T. (2003). Prediction of the prototype of the human toll-like receptor gene family from the pufferfish, *Fugu rubripes*, genome. Immunogenetics.

[34] Hwang S.D., Ohtani M., Hikima J.-I., Jung T.S., Kondo H., Hirono I., Aoki T. (2012). Molecular cloning and characterization of toll-like receptor 3 in Japanese flounder, *Paralichthys olivaceus*. Dev. Comp. Immunol..

[35] Avunje S., Kim W.-S., Park C.-S., Oh M.-J., Jung S.-J. (2011). Toll-like receptors and interferon associated immune factors in viral haemorrhagic septicaemia virus-infected olive flounder (*Paralichthys olivaceus*). Fish Shellfish Immunol..

[36] Hwang S.D., Fuji K., Takano T., Sakamoto T., Kondo H., Hirono I., Aoki T. (2011). Linkage mapping of toll-like receptors (TLRs) in Japanese flounder, *Paralichthys olivaceus*. Mar. Biotechnol..

[37] Takano T., Kondo H., Hirono I., Endo M., Saito-Taki T., Aoki T. (2007). Molecular cloning and characterization of toll-like receptor 9 in Japanese flounder, *Paralichthys olivaceus*. Mol. Immunol..

[38] Hirono I., Takami M., Miyata M., Miyazaki T., Han H.-J., Takano T., Endo M., Aoki T. (2004). Characterization of gene structure and expression of two toll-like receptors from Japanese flounder, *Paralichthys olivaceus*. Immunogenetics.

[39] Ohtani M., Hikima J.-I., Kondo H., Hirono I., Jung T.-S., Aoki T. (2011). Characterization and antiviral function of a cytosolic sensor gene, MDA5, in Japanese flounder, *Paralichthys olivaceus*. Dev. Comp. Immunol..

[40] Ohtani M., Hikima J.-I., Kondo H., Hirono I., Jung T.-S., Aoki T. (2010). Evolutional conservation of molecular structure and antiviral function of a viral RNA receptor, LGP2, in Japanese flounder, *Paralichthys olivaceus*. J. Immunol..

[41] Quynh N.T., Hikima J.-I., Kim Y.-R., Fagutao F.F., Kim M.S., Aoki T., Jung T.S. (2015). The cytosolic sensor, DDX41, activates antiviral and inflammatory immunity in response to stimulation with double-stranded DNA adherent cells of the olive flounder, *Paralichthys olivaceus*. Fish Shellfish Immunol..

[42] Huang X.-N., Wang Z.-Y., Yao C.-L. (2011). Characterization of toll-like receptor 3 gene in large yellow croaker, *Pseudosciaena crocea*. Fish Shellfish Immunol..

[43] Qian T., Wang K., Mu Y., Ao J., Chen X. (2013). Molecular characterization and expression analysis of TLR 7 and TLR 8 homologs in large yellow croaker (*Pseudosciaena crocea*). Fish Shellfish Immunol..

[44] Yao C.-L., Kong P., Wang Z.-Y., Ji P.-F., Cai M.-Y., Liu X.-D., Han X.-Z. (2008). Cloning and expression analysis of two alternative splicing toll-like receptor 9 isoforms A and B in large yellow croaker, *Pseudosciaena crocea*. Fish Shellfish Immunol..

[45] Xiao X., Qin Q., Chen X. (2011). Molecular characterization of a toll-like receptor 22 homologue in large yellow croaker (*Pseudosciaena crocea*) and promoter activity analysis of its 5'-flanking sequence. Fish Shellfish Immunol..

[46] Wang X., Wang K., Nie P., Chen X., Ao J. (2014). Establishment and characterization of a head kidney cell line from large yellow croaker *Pseudosciaena crocea*. J. Fish Biol..

[47] He J., Liu H., Wu C. (2014). Identification of SCARA3, SCARA5 and MARCO of class A scavenger receptor-like family in *Pseudosciaena crocea*. Fish Shellfish Immunol..

[48] Rodriguez M., Wiens G., Purcell M., Palti Y. (2005). Characterization of toll-like receptor 3 gene in rainbow trout (*Oncorhynchus mykiss*). Immunogenetics.

[49] Palti Y., Gahr S.A., Purcell M.K., Hadidi S., Rexroad C.E., Wiens G.D. (2010). Identification, characterization and genetic mapping of TLR7, TLR8a1 and TLR8a2 genes in rainbow trout (*Oncorhynchus mykiss*). Dev. Comp. Immunol..

[50] Ortega-Villaizan M., Chico V., Falco A., Perez L., Coll J., Estepa A. (2009). The rainbow trout TLR9 gene and its role in the immune responses elicited by a plasmid encoding the glycoprotein G of the viral haemorrhagic septicaemia rhabdovirus (VHSV). Mol. Immunol..

[51] Chang M., Collet B., Nie P., Lester K., Campbell S., Secombes C.J., Zou J. (2011). Expression and functional characterization of the RIG-I-like receptors MDA5 and LGP2 in Rainbow trout (*Oncorhynchus mykiss*). J. Virol..

[52] Poynter S.J., Weleff J., Soares A.B., Dewitte-Orr S.J. (2015). Class-A scavenger receptor function and expression in the rainbow trout (*Oncorhynchus mykiss*) epithelial cell lines RTgutGC and RTgill-W1. Fish Shellfish Immunol..

[53] Meijer A.H., Krens S.G., Rodriguez I.A.M., He S., Bitter W., Snaar-Jagalska B.E., Spaink H.P. (2004). Expression analysis of the toll-like receptor and TIR domain adaptor families of zebrafish. Mol. Immunol..

[54] Nie L., Zhang Y.-S., Dong W.-R., Xiang L.-X., Shao J.-Z. (2015). Involvement of zebrafish RIG-I in NF-κB and IFN signaling pathways: Insights into functional conservation of RIG-I in antiviral innate immunity. Dev. Comp. Immunol..

[55] Chen W.Q., Hu Y.W., Zou P.F., Ren S.S., Nie P., Chang M.X. (2015). MAVS splicing variants contribute to the induction of interferon and interferon-stimulated genes mediated by RIG-I-like receptors. Dev. Comp. Immunol..

[56] Fukuda M., Ohtani K., Jang S.-J., Yoshizaki T., Mori K.-I., Motomura W., Yoshida I., Suzuki Y., Kohgo Y., Wakamiya N. (2011). Molecular cloning and functional analysis of scavenger receptor zebrafish Cl-P1. Biochim. Biophys. Acta.

[57] Benard E.L., Roobol S.J., Spaink H.P., Meijer A.H. (2014). Phagocytosis of mycobacteria by zebrafish macrophages is dependent on the scavenger receptor marco, a key control factor of pro-inflammatory signalling. Dev. Comp. Immunol..

[58] Bell J.K., Mullen G.E., Leifer C.A., Mazzoni A., Davies D.R., Segal D.M. (2003). Leucine-rich repeats and pathogen recognition in toll-like receptors. Trends Immunol..

[59] Palti Y. (2011). Toll-like receptors in bony fish: From genomics to function. Dev. Comp. Immunol..

[60] Zhang J., Kong X., Zhou C., Li L., Nie G., Li X. (2014). Toll-like receptor recognition of bacteria in fish: Ligand specificity and signal pathways. Fish Shellfish Immunol..

[61] Moresco E.M.Y., LaVine D., Beutler B. (2011). Toll-like receptors. Curr. Biol..

[62] Li X.-D., Chen Z.J. (2012). Sequence specific detection of bacterial 23S ribosomal RNA by TLR13. Elife.

[63] Yeh D.-W., Liu Y.-L., Lo Y.-C., Yuh C.-H., Yu G.-Y., Lo J.-F., Luo Y., Xiang R., Chuang T.-H. (2013). Toll-like receptor 9 and 21 have different ligand recognition profiles and cooperatively mediate activity of CpG-oligodeoxynucleotides in zebrafish. PNAS.

[64] Matsuo A., Oshiumi H., Tsujita T., Mitani H., Kasai H., Yoshimizu M., Matsumoto M., Seya T. (2008). Teleost TLR22 recognizes RNA duplex to induce IFN and protect cells from birnaviruses. J. Immunol..

[65] Barton G.M., Kagan J.C. (2009). A cell biological view of Toll-like receptor function: Regulation through compartmentalization. Nat. Rev. Immunol..

[66] Matsumoto M., Kikkawa S., Kohase M., Miyake K., Seya T. (2002). Establishment of a monoclonal antibody against human toll-like receptor 3 that blocks double-stranded RNA-mediated signaling. Biochem. Biophys. Res. Commun..

[67] O’Neill L.A., Golenbock D., Bowie A.G. (2013). The history of Toll-like receptors—Redefining innate immunity. Nat. Rev. Immunol..

[68] Leifer C.A., Kennedy M.N., Mazzoni A., Lee C., Kruhlak M.J., Segal D.M. (2004). TLR9 is localized in the endoplasmic reticulum prior to stimulation. J. Immunol..

[69] Brownlie R., Zhu J., Allan B., Mutwiri G.K., Babiuk L.A., Potter A., Griebel P. (2009). Chicken TLR21 acts as a functional homologue to mammalian TLR9 in the recognition of CpG oligodeoxynucleotides. Mol. Immunol..

[70] Jørgensen J., Zou J., Johansen A., Secombes C. (2001). Immunostimulatory CpG oligodeoxynucleotides stimulate expression of IL-1β and interferon-like cytokines in rainbow trout macrophages via a chloroquine-sensitive mechanism. Fish Shellfish Immunol..

[71] Zhou Z.-X., Sun L. (2015). Immune effects of R848: Evidences that suggest an essential role of TLR7/8-induced, MyD88-and NF-κB-dependent signaling in the antiviral immunity of Japanese flounder (*Paralichthys olivaceus*). Dev. Comp. Immunol..

[72] DeWitte-Orr S.J., Mossman K.L. (2010). dsRNA and the innate antiviral immune response. Future Virol..

[73] Józefowski S., Sulahian T.H., Arredouani M., Kobzik L. (2006). Role of scavenger receptor MARCO in macrophage responses to CpG oligodeoxynucleotides. J. Leukocyte Biol..

[74] Delgado M., Deretic V. (2009). Toll-like receptors in control of immunological autophagy. Cell Death Differ..

[75] Kawasaki T., Kawai T. (2014). Toll-like receptor signaling pathways. Front. Immunol..

[76] Samanta M., Basu M., Swain B., Panda P., Jayasankar P. (2013). Molecular cloning and characterization of toll-like receptor 3, and inductive expression analysis of type I IFN, Mx and pro-inflammatory cytokines in the Indian carp, rohu (*Labeo rohita*). Mol. Biol. Rep..

[77] Phelan P.E., Mellon M.T., Kim C.H. (2005). Functional characterization of full-length TLR3, IRAK-4, and TRAF6 in zebrafish (*Danio rerio*). Mol. Immunol..

[78] Lin K., Ge H., Lin Q., Wu J., He L., Fang Q., Zhou C., Sun M., Huang Z. (2013). Molecular characterization and functional analysis of toll-like receptor 3 gene in orange-spotted grouper (*Epinephelus coioides*). Gene.

[79] Zhou Z.-X., Zhang B.-C., Sun L. (2014). Poly (I:C) induces antiviral immune responses in Japanese flounder (*Paralichthys olivaceus*) that require TLR3 and MDA5 and is negatively regulated by MyD88. PLoS ONE.

[80] Heil F., Hemmi H., Hochrein H., Ampenberger F., Kirschning C., Akira S., Lipford G., Wagner H., Bauer S. (2004). Species-specific recognition of single-stranded RNA via toll-like receptor 7 and 8. Science.

[81] Gantier M.P., Tong S., Behlke M.A., Xu D., Phipps S., Foster P.S., Williams B.R. (2008). TLR7 is involved in sequence-specific sensing of single-stranded RNAs in human macrophages. J. Immunol..

[82] Hemmi H., Kaisho T., Takeuchi O., Sato S., Sanjo H., Hoshino K., Horiuchi T., Tomizawa H., Takeda K., Akira S. (2002). Small anti-viral compounds activate immune cells via the TLR7 MyD88-dependent signaling pathway. Nat. Immunol..

[83] Gorden K.B., Gorski K.S., Gibson S.J., Kedl R.M., Kieper W.C., Qiu X., Tomai M.A., Alkan S.S., Vasilakos J.P. (2005). Synthetic TLR agonists reveal functional differences between human TLR7 and TLR8. J. Immunol..

[84] Chen X., Wang Q., Yang C., Rao Y., Li Q., Wan Q., Peng L., Wu S., Su J. (2013). Identification, expression profiling of a grass carp TLR8 and its inhibition leading to the resistance to reovirus in CIK cells. Dev. Comp. Immunol..

[85] Lund J., Sato A., Akira S., Medzhitov R., Iwasaki A. (2003). Toll-like receptor 9-mediated recognition of herpes simplex virus-2 by plasmacytoid dendritic cells. J. Exp. Med..

[86] Kawai T., Akira S. (2006). TLR signaling. Cell Death Differ..

[87] Roberts T.L., Sweet M.J., Hume D.A., Stacey K.J. (2005). Cutting edge: Species-specific TLR9-mediated recognition of CpG and non-CpG phosphorothioate-modified oligonucleotides. J. Immunol..

[88] Zelenay S., Elías F., Fló J. (2003). Immunostimulatory effects of plasmid DNA and synthetic oligodeoxynucleotides. Eur. J. Immunol..

[89] Cuesta A., Esteban M., Meseguer J. (2008). The expression profile of TLR9 mRNA and CpG ODNs immunostimulatory actions in the teleost gilthead seabream points to a major role of lymphocytes. Cell. Mol. Life Sci..

[90] Byadgi O., Puteri D., Lee Y.-H., Lee J.-W., Cheng T.-C. (2014). Identification and expression analysis of cobia (*Rachycentron canadum*) toll-like receptor 9 gene. Fish Shellfish Immunol..

[91] Strandskog G., Villoing S., Iliev D.B., Thim H.L., Christie K.E., Jørgensen J.B. (2011). Formulations combining CpG containing oliogonucleotides and poly I:C enhance the magnitude of immune responses and protection against pancreas disease in Atlantic salmon. Dev. Comp. Immunol..

[92] Jørgensen J.B., Johansen A., Stenersen B., Sommer A.-I. (2001). CpG oligodeoxynucleotides and plasmid DNA stimulate Atlantic salmon (*Salmo salar* L.) leucocytes to produce supernatants with antiviral activity. Dev. Comp. Immunol..

[93] Iliev D.B., Skjaeveland I., Jørgensen J.B. (2013). CpG oligonucleotides bind TLR9 and RRM-containing proteins in Atlantic salmon (*Salmo salar*). BMC Immunol..

[94] Broz P., Monack D.M. (2013). Newly described pattern recognition receptors team up against intracellular pathogens. Nat. Rev. Immunol..

[95] Shi Z., Cai Z., Sanchez A., Zhang T., Wen S., Wang J., Zhang D. (2011). A novel Toll-like receptor that recognizes vesicular stomatitis virus. J. Biol. Chem..

[96] Leong J.S., Jantzen S.G., von Schalburg K.R., Cooper G.A., Messmer A.M., Liao N.Y., Munro S., Moore R., Holt R.A., Jones S.J. (2010). *Salmo salar* and *Esox lucius* full-length cDNA sequences reveal changes in evolutionary pressures on a post-tetraploidization genome. BMC Genomics.

[97] Keestra A.M., de Zoete M.R., Bouwman L.I., van Putten J.P. (2010). Chicken TLR21 is an innate CpG DNA receptor distinct from mammalian TLR9. J. Immunol..

[98] Sundaram A.Y., Kiron V., Dopazo J., Fernandes J.M. (2012). Diversification of the expanded teleost-specific toll-like receptor family in Atlantic cod, *Gadus morhua*. BMC Evol. Biol..

[99] Priyathilaka T.T., Elvitigala D.A.S., Whang I., Lim B.-S., Jeong H.-B., Yeo S.-Y., Choi C.Y., Lee J. (2014). Molecular characterization and transcriptional analysis of non-mammalian type toll like receptor (TLR21) from Rock bream (*Oplegnathus fasciatus*). Gene.

[100] Liu L., Botos I., Wang Y., Leonard J.N., Shiloach J., Segal D.M., Davies D.R. (2008). Structural basis of toll-like receptor 3 signaling with double-stranded RNA. Science.

[101] Hu G.-B., Zhang S.-F., Yang X., Liu D.-H., Liu Q.-M., Zhang S.-C. (2015). Cloning and expression analysis of a toll-like receptor 22 (TLR22) gene from turbot, *Scophthalmus maximus*. Fish Shellfish Immunol..

[102] Muñoz I., Sepulcre M.P., Meseguer J., Mulero V. (2014). Toll-like receptor 22 of gilthead seabream, *Sparus aurata*: Molecular cloning, expression profiles and post-transcriptional regulation. Dev. Comp. Immunol..

[103] Samanta M., Swain B., Basu M., Mahapatra G., Sahoo B.R., Paichha M., Lenka S.S., Jayasankar P. (2014). Toll-like receptor 22 in *Labeo rohita*: Molecular cloning, characterization, 3D modeling, and expression analysis following ligands stimulation and bacterial infection. Appl. Biochem. Biotech..

[104] Su J., Huang T., Yang C., Zhang R. (2011). Molecular cloning, characterization and expression analysis of interferon-β promoter stimulator 1 (IPS-1) gene from grass carp *Ctenopharyngodon idella*. Fish Shellfish Immunol..

[105] Takano T., Kondo H., Hirono I., Saito-Taki T., Endo M., Aoki T. (2006). Identification and characterization of a myeloid differentiation factor 88 (MyD88) cDNA and gene in Japanese flounder, *Paralichthys olivaceus*. Dev. Comp. Immunol..

[106] Yao C.-L., Kong P., Wang Z.-Y., Ji P.-F., Liu X.-D., Cai M.-Y., Han X.-Z. (2009). Molecular cloning and expression of MyD88 in large yellow croaker, *Pseudosciaena crocea*. Fish Shellfish Immunol..

[107] Rebl A., Goldammer T., Fischer U., Köllner B., Seyfert H.-M. (2009). Characterization of two key molecules of teleost innate immunity from rainbow trout (*Oncorhynchus mykiss*): MyD88 and SAA. Vet. Immunol. Immunopathol..

[108] Van der Sar A.M., Stockhammer O.W., van der Laan C., Spaink H.P., Bitter W., Meijer A.H. (2006). MyD88 innate immune function in a zebrafish embryo infection model. Infect. Immun..

[109] Foley J.F. (2015). TRAF6 phosphorylation inhibits inflammation. Sci. Signal..

[110] Li Y.-W., Mo X.-B., Zhou L., Li X., Dan X.-M., Luo X.-C., Li A.-X. (2014). Identification of IRAK-4 in grouper (*Epinephelus coioides*) that impairs MyD88-dependent NF-κB activation. Dev. Comp. Immunol..

[111] Basu M., Swain B., Sahoo B.R., Maiti N.K., Samanta M. (2012). Induction of toll-like receptor (TLR) 2, and MyD88-dependent TLR-signaling in response to ligand stimulation and bacterial infections in the Indian major carp, mrigal (*Cirrhinus mrigala*). Mol. Biol. Rep..

[112] Iliev D.B., Sobhkhez M., Fremmerlid K., Jorgensen J.B. (2011). MyD88 interacts with interferon regulatory factor (IRF)3 and IRF7 in Atlantic salmon (*Salmo salar*): Transgenic SsMyD88 modulates the IRF-induced type I interferon response and accumulates in aggresomes. J. Biol. Chem..

[113] Baoprasertkul P., Peatman E., Somridhivej B., Liu Z. (2006). Toll-like receptor 3 and TICAM genes in catfish: Species-specific expression profiles following infection with *Edwardsiella ictaluri*. Immunogenetics.

[114] Fan S., Chen S., Liu Y., Lin Y., Liu H., Guo L., Lin B., Huang S., Xu A. (2008). Zebrafish TRIF, a Golgi-localized protein, participates in IFN induction and NF-κB activation. J. Immunol..

[115] Zhang Y.-B., Gui J.-F. (2012). Molecular regulation of interferon antiviral response in fish. Dev. Comp. Immunol..

[116] Seya T., Matsumoto M., Ebihara T., Oshiumi H. (2009). Functional evolution of the TICAM-1 pathway for extrinsic RNA sensing. Immunol. Rev..

[117] Takeuchi O., Akira S. (2008). MDA5/RIG-I and virus recognition. Curr. Opin. Immunol..

[118] Hornung V., Ellegast J., Kim S., Brzózka K., Jung A., Kato H., Poeck H., Akira S., Conzelmann K.-K., Schlee M. (2006). 5'-triphosphate RNA is the ligand for RIG-I. Science.

[119] Goubau D., Schlee M., Deddouche S., Pruijssers A.J., Zillinger T., Goldeck M., Schuberth C., van der Veen A.G., Fujimura T., Rehwinkel J. (2014). Antiviral immunity via RIG-I-mediated recognition of RNA bearing 5'-diphosphates. Nature.

[120] Kato H., Takahasi K., Fujita T. (2011). RIG-I-like receptors: Cytoplasmic sensors for non-self RNA. Immunol. Rev..

[121] Kato H., Takeuchi O., Sato S., Yoneyama M., Yamamoto M., Matsui K., Uematsu S., Jung A., Kawai T., Ishii K.J. (2006). Differential roles of MDA5 and RIG-I helicases in the recognition of RNA viruses. Nature.

[122] Zou P.F., Chang M.X., Li Y., Zhang S.H., Fu J.P., Chen S.N., Nie P. (2015). Higher antiviral response of RIG-I through enhancing RIG-I/MAVs-mediated signaling by its long insertion variant in zebrafish. Fish Shellfish Immunol..

[123] Chen H.-Y., Liu W., Wu S.-Y., Chiou P.P., Li Y.-H., Chen Y.-C., Lin G.-H., Lu M.-W., Wu J.-L. (2015). RIG-I specifically mediates group II type I IFN activation in nervous necrosis virus infected zebrafish cells. Fish Shellfish Immunol..

[124] Wang W., Asim M., Yi L., Hegazy A.M., Hu X., Zhou Y., Ai T., Lin L. (2015). Abortive infection of snakehead fish vesiculovirus in ZF4 cells was associated with the RLRs pathway activation by viral replicative intermediates. Int. J. Mol. Sci..

[125] Wang B., Zhang Y.-B., Liu T.-K., Shi J., Sun F., Gui J.-F. (2014). Fish viperin exerts a conserved antiviral function through RLR-triggered IFN signaling pathway. Dev. Comp. Immunol..

[126] Satoh T., Kato H., Kumagai Y., Yoneyama M., Sato S., Matsushita K., Tsujimura T., Fujita T., Akira S., Takeuchi O. (2010). LGP2 is a positive regulator of RIG-I–and MDA5-mediated antiviral responses. PNAS.

[127] Yoneyama M., Fujita T. (2008). Structural mechanism of RNA recognition by the RIG-I-like receptors. Immunity.

[128] Holland J., Bird S., Williamson B., Woudstra C., Mustafa A., Wang T., Zou J., Blaney S., Collet B., Secombes C. (2008). Molecular characterization of IRF3 and IRF7 in rainbow trout, Oncorhynchus mykiss: Functional analysis and transcriptional modulation. Mol. Immunol..

[129] Loo Y.-M., Fornek J., Crochet N., Bajwa G., Perwitasari O., Martinez-Sobrido L., Akira S., Gill M.A., García-Sastre A., Katze M.G. (2008). Distinct RIG-I and MDA5 signaling by RNA viruses in innate immunity. J. Virol..

[130] Bhat A., Paria A., Deepika A., Sreedharan K., Makesh M., Bedekar M.K., Purushothaman C., Rajendran K. (2015). Molecular cloning, characterisation and expression analysis of melanoma differentiation associated gene 5 (MDA5) of green chromide, *Etroplus suratensis*. Gene.

[131] Zou P.F., Chang M.X., Xue N.N., Liu X.Q., Li J.H., Fu J.P., Chen S.N., Nie P. (2014). Melanoma differentiation-associated gene 5 in zebrafish provoking higher interferon-promoter activity through signalling enhancing of its shorter splicing variant. Immunology.

[132] Yoneyama M., Onomoto K., Jogi M., Akaboshi T., Fujita T. (2015). Viral RNA detection by RIG-I-like receptors. Curr. Opin. Immunol..

[133] Bruns A.M., Leser G.P., Lamb R.A., Horvath C.M. (2014). The innate immune sensor LGP2 activates antiviral signaling by regulating MDA5-RNA interaction and filament assembly. Mol. Cell.

[134] Venkataraman T., Valdes M., Elsby R., Kakuta S., Caceres G., Saijo S., Iwakura Y., Barber G.N. (2007). Loss of DEXD/H box RNA helicase LGP2 manifests disparate antiviral responses. J. Immunol..

[135] Chen X., Yang C., Su J., Rao Y., Gu T. (2015). LGP2 plays extensive roles in modulating innate immune responses in *Ctenopharyngodon idella* kidney (cik) cells. Dev. Comp. Immunol..

[136] Cao X., Chen J., Cao Y., Nie G., Wan Q., Wang L., Su J. (2015). Identification and expression of the laboratory of genetics and physiology 2 gene in common carp *Cyprinus carpio*. J. Fish Biol..

[137] Lauksund S., Svingerud T., Bergan V., Robertsen B. (2009). Atlantic salmon IPS-1 mediates induction of IFNa1 and activation of NF-κB and localizes to mitochondria. Dev. Comp. Immunol..

[138] Simora R.M.C., Ohtani M., Hikima J.-I., Kondo H., Hirono I., Jung T.S., Aoki T. (2010). Molecular cloning and antiviral activity of IFN-β promoter stimulator-1 (IPS-1) gene in Japanese flounder, *Paralichthys olivaceus*. Fish Shellfish Immunol..

[139] Zhang J., Zhang Y.-B., Wu M., Wang B., Chen C., Gui J.-F. (2014). Fish MAVs is involved in RLR pathway-mediated IFN response. Fish Shellfish Immunol..

[140] Taniguchi T., Ogasawara K., Takaoka A., Tanaka N. (2001). IRF family of transcription factors as regulators of host defense. Annu. Rev. Immunol..

[141] Honda K., Takaoka A., Taniguchi T. (2006). Type I interferon gene induction by the interferon regulatory factor family of transcription factors. Immunity.

[142] Sun F., Zhang Y.-B., Liu T.-K., Gan L., Yu F.-F., Liu Y., Gui J.-F. (2010). Characterization of fish IRF3 as an IFN-inducible protein reveals evolving regulation of IFN response in vertebrates. J. Immunol..

[143] Takaoka A., Wang Z., Choi M.K., Yanai H., Negishi H., Ban T., Lu Y., Miyagishi M., Kodama T., Honda K. (2007). DAI (DLM-1ZBP1) is a cytosolic DNA sensor and an activator of innate immune response. Nature.

[144] Hornung V., Ablasser A., Charrel-Dennis M., Bauernfeind F., Horvath G., Caffrey D.R., Latz E., Fitzgerald K.A. (2009). AIM2 recognizes cytosolic dsDNA and forms a caspase-1-activating inflammasome with ASC. Nature.

[145] Fernandes-Alnemri T., Yu J.-W., Datta P., Wu J., Alnemri E.S. (2009). AIM2 activates the inflammasome and cell death in response to cytoplasmic DNA. Nature.

[146] Roberts T.L., Idris A., Dunn J.A., Kelly G.M., Burnton C.M., Hodgson S., Hardy L.L., Garceau V., Sweet M.J., Ross I.L. (2009). HIN-200 proteins regulate caspase activation in response to foreign cytoplasmic DNA. Science.

[147] Ablasser A., Bauernfeind F., Hartmann G., Latz E., Fitzgerald K.A., Hornung V. (2009). RIG-I-dependent sensing of poly (dA: dT) through the induction of an RNA polymerase III-transcribed RNA intermediate. Nat. Immunol..

[148] Yang P., An H., Liu X., Wen M., Zheng Y., Rui Y., Cao X. (2010). The cytosolic nucleic acid sensor LRRFIP1 mediates the production of type I interferon via a [beta]-catenin-dependent pathway. Nat. Immunol..

[149] Zhang Z., Yuan B., Bao M., Lu N., Kim T., Liu Y.-J. (2011). The helicase DDX41 senses intracellular DNA mediated by the adaptor STING in dendritic cells. Nat. Immunol..

[150] Sun L., Wu J., Du F., Chen X., Chen Z.J. (2013). Cyclic GMP-AMP synthase is a cytosolic DNA sensor that activates the type I interferon pathway. Science.

[151] Unterholzner L., Keating S.E., Baran M., Horan K.A., Jensen S.B., Sharma S., Sirois C.M., Jin T., Latz E., Xiao T.S. (2010). IFI16 is an innate immune sensor for intracellular DNA. Nat. Immunol..

[152] Kerur N., Veettil M.V., Sharma-Walia N., Bottero V., Sadagopan S., Otageri P., Chandran B. (2011). IFI16 acts as a nuclear pathogen sensor to induce the inflammasome in response to Kaposi Sarcoma-associated herpesvirus infection. Cell Host Microbe.

[153] DeFilippis V.R., Alvarado D., Sali T., Rothenburg S., Früh K. (2010). Human cytomegalovirus induces the interferon response via the DNA sensor ZBP1. J. Virol..

[154] Rathinam V.A., Jiang Z., Waggoner S.N., Sharma S., Cole L.E., Waggoner L., Vanaja S.K., Monks B.G., Ganesan S., Latz E. (2010). The AIM2 inflammasome is essential for host defense against cytosolic bacteria and DNA viruses. Nat. Immunol..

[155] Biacchesi S., Mérour E., Lamoureux A., Bernard J., Brémont M. (2012). Both STING and MAVS fish orthologs contribute to the induction of interferon mediated by RIG-I. PLoS ONE.

[156] Burdette D.L., Vance R.E. (2013). STING and the innate immune response to nucleic acids in the cytosol. Nat. Immunol..

[157] Ishikawa H., Ma Z., Barber G.N. (2009). STING regulates intracellular DNA-mediated, type I interferon-dependent innate immunity. Nature.

[158] Jones J.W., Kayagaki N., Broz P., Henry T., Newton K., O’Rourke K., Chan S., Dong J., Qu Y., Roose-Girma M. (2010). Absent in melanoma 2 is required for innate immune recognition of *Francisella tularensis*. PNAS.

[159] Prantner D., Darville T., Nagarajan U.M. (2010). Stimulator of IFN gene is critical for induction of IFN-β during *Chlamydia muridarum* infection. J. Immunol..

[160] Jin L., Hill K.K., Filak H., Mogan J., Knowles H., Zhang B., Perraud A.-L., Cambier J.C., Lenz L.L. (2011). MPYS is required for IFN response factor 3 activation and type I IFN production in the response of cultured phagocytes to bacterial second messengers cyclic-di-AMP and cyclic-di-GMP. J. Immunol..

[161] Sauer J.-D., Sotelo-Troha K., von Moltke J., Monroe K.M., Rae C.S., Brubaker S.W., Hyodo M., Hayakawa Y., Woodward J.J., Portnoy D.A. (2011). The *N*-ethyl-*N*-nitrosourea-induced Goldenticket mouse mutant reveals an essential function of STING in the *in vivo* interferon response to *Listeria monocytogenes* and cyclic dinucleotides. Infect. Immun..

[162] Abe T., Harashima A., Xia T., Konno H., Konno K., Morales A., Ahn J., Gutman D., Barber G.N. (2013). STING recognition of cytoplasmic DNA instigates cellular defense. Mol. Cell.

[163] Sun F., Zhang Y.-B., Liu T.-K., Shi J., Wang B., Gui J.-F. (2011). Fish MITA serves as a mediator for distinct fish IFN gene activation dependent on IRF3 or IRF7. J. Immunol..

[164] Hornung V., Latz E. (2010). Intracellular DNA recognition. Nat. Rev. Immunol..

[165] Sullivan C., Postlethwait J.H., Lage C.R., Millard P.J., Kim C.H. (2007). Evidence for evolving Toll-IL-1 receptor-containing adaptor molecule function in vertebrates. J. Immunol..

[166] Chi H., Zhang Z., Bøgwald J., Zhan W., Dalmo R.A. (2011). Cloning, expression analysis and promoter structure of TBK1 (tank-binding kinase 1) in Atlantic cod (*Gadus morhua* L.). Fish Shellfish Immunol..

[167] Feng H., Liu H., Kong R., Wang L., Wang Y., Hu W., Guo Q. (2011). Expression profiles of carp IRF-3/-7 correlate with the up-regulation of RIG-I/MAVS/TRAF3/TBK1, four pivotal molecules in RIG-I signaling pathway. Fish Shellfish Immunol..

[168] Goldstein J.L., Ho Y., Basu S.K., Brown M.S. (1979). Binding site on macrophages that mediates uptake and degradation of acetylated low density lipoprotein, producing massive cholesterol deposition. PNAS.

[169] DeWitte-Orr S.J., Collins S.E., Bauer C.M., Bowdish D.M., Mossman K.L. (2010). An accessory to the “trinity”: SR-As are essential pathogen sensors of extracellular dsRNA, mediating entry and leading to subsequent type I IFN responses. PLoS Pathog..

[170] Pearson A.M. (1996). Scavenger receptors in innate immunity. Curr. Opin. Immunol..

[171] Roach J.C., Glusman G., Rowen L., Kaur A., Purcell M.K., Smith K.D., Hood L.E., Aderem A. (2005). The evolution of vertebrate toll-like receptors. PNAS.

[172] Doi T., Higashino K.-I., Kurihara Y., Wada Y., Miyazaki T., Nakamura H., Uesugi S., Imanishi T., Kawabe Y., Itakura H. (1993). Charged collagen structure mediates the recognition of negatively charged macromolecules by macrophage scavenger receptors. J. Biol. Chem..

[173] Ojala J.R., Pikkarainen T., Tuuttila A., Sandalova T., Tryggvason K. (2007). Crystal structure of the cysteine-rich domain of scavenger receptor MARCO reveals the presence of a basic and an acidic cluster that both contribute to ligand recognition. J. Biol. Chem..

[174] Holmskov U., Malhotra R., Sim R.B., Jensenius J.C. (1994). Collectins: Collagenous C-type lectins of the innate immune defense system. Immunol. Today.

[175] Martínez V.G., Moestrup S.K., Holmskov U., Mollenhauer J., Lozano F. (2011). The conserved scavenger receptor cysteine-rich superfamily in therapy and diagnosis. Pharmacol. Rev..

[176] Brown M.S., Goldstein J.L. (1983). Lipoprotein metabolism in the macrophage: Implications for cholesterol deposition in atherosclerosis. Annu. Rev. Biochem..

[177] Dumont E., Rallière C., Rescan P.-Y. (2008). Identification of novel genes including Dermo-1, a marker of dermal differentiation, expressed in trout somitic external cells. J. Exp. Biol..

[178] Meng Z., Zhang X.-Y., Guo J., Xiang L.-X., Shao J.-Z. (2012). Scavenger receptor in fish is a lipopolysaccharide recognition molecule involved in negative regulation of NF-κB activation by competing with TNF receptor-associated factor 2 recruitment into the TNF-α signaling pathway. J. Immunol..

[179] Martin-Armas M., Zykova S., Smedsrød B. (2008). Effects of CpG-oligonucleotides, poly I:C and LPS on Atlantic cod scavenger endothelial cells (SEC). Dev. Comp. Immunol..

[180] Kaur H., Jaso-Friedmann L., Evans D.L. (2003). Identification of a scavenger receptor homologue on nonspecific cytotoxic cells and evidence for binding to oligodeoxyguanosine. Fish Shellfish Immunol..

[181] Frøystad M.K., Rode M., Berg T., Gjøen T. (1998). A role for scavenger receptors in phagocytosis of protein-coated particles in rainbow trout head kidney macrophages. Dev. Comp. Immunol..

[182] Hsu H.-Y., Hajjar D.P., Khan K.F., Falcone D.J. (1998). Ligand binding to macrophage scavenger receptor-A induces urokinase-type plasminogen activator expression by a protein kinase-dependent signaling pathway. J. Biol. Chem..

[183] Whitman S.C., Daugherty A., Post S.R. (2000). Regulation of acetylated low density lipoprotein uptake in macrophages by pertussis toxin-sensitive G proteins. J. Lipid Res..

[184] Hsu H.-Y., Chiu S.-L., Wen M.-H., Chen K.-Y., Hua K.-F. (2001). Ligands of macrophage scavenger receptor induce cytokine expression via differential modulation of protein kinase signaling pathways. J. Biol. Chem..

[185] Coller S.P., Paulnock D.M. (2001). Signaling pathways initiated in macrophages after engagement of type A scavenger receptors. J. Leukocyte Biol..

[186] Kim W.S., Ordija C.M., Freeman M.W. (2003). Activation of signaling pathways by putative scavenger receptor class A (SR-A) ligands requires CD14 but not SR-A. Biochem. Biophys. Res. Comm..

[187] Murphy J.E., Tedbury P.R., Homer-Vanniasinkam S., Walker J.H., Ponnambalam S. (2005). Biochemistry and cell biology of mammalian scavenger receptors. Atherosclerosis.

[188] Zou J., Secombes C.J. (2011). Teleost fish interferons and their role in immunity. Dev. Comp. Immunol..

[189] Fensterl V., Sen G.C. (2009). Interferons and viral infections. Biofactors.

[190] González-Navajas J.M., Lee J., David M., Raz E. (2012). Immunomodulatory functions of type I interferons. Nat. Rev. Immunol..

[191] Goodbourn S., Didcock L., Randall R. (2000). Interferons: Cell signalling, immune modulation, antiviral response and virus countermeasures. J. Gen. Virol..

[192] Parhi J., Mukherjee S., Saxena G., Sahoo L., Makesh M. (2014). Molecular characterization and expression of type-I interferon gene in *Labeo rohita*. Mol. Biol. Rep..

[193] Ohtani M., Hikima J.-I., Hwang S.D., Morita T., Suzuki Y., Kato G., Kondo H., Hirono I., Jung T.-S., Aoki T. (2012). Transcriptional regulation of type I interferon gene expression by interferon regulatory factor-3 in Japanese flounder, *Paralichthys olivaceus*. Dev. Comp. Immunol..

[194] Wan Q., Wicramaarachchi W.N., Whang I., Lim B.-S., Oh M.-J., Jung S.-J., Kim H.C., Yeo S.-Y., Lee J. (2012). Molecular cloning and functional characterization of two duplicated two-cysteine containing type I interferon genes in rock bream *Oplegnathus fasciatus*. Fish Shellfish Immunol..

[195] Li D., Tan W., Ma M., Yu X., Lai Q., Wu Z., Lin G., Hu C. (2012). Molecular characterization and transcription regulation analysis of type I IFN gene in grass carp (*Ctenopharyngodon idella*). Gene.

[196] Adamek M., Rakus K.Ł., Chyb J., Brogden G., Huebner A., Irnazarow I., Steinhagen D. (2012). Interferon type I responses to virus infections in carp cells: *In vitro* studies on Cyprinid herpesvirus 3 and Rhabdovirus carpio infections. Fish Shellfish Immunol..

[197] Haller O., Staeheli P., Kochs G. (2007). Interferon-induced Mx proteins in antiviral host defense. Biochimie.

[198] Leong J.A.C., Trobridge G.D., Kim C.H., Johnson M., Simon B. (1998). Interferon-inducible Mx proteins in fish. Immunol. Rev..

[199] Pavlovic J., Zürcher T., Haller O., Staeheli P. (1990). Resistance to influenza virus and vesicular stomatitis virus conferred by expression of human MxA protein. J. Virol..

[200] Staeheli P., Haller O., Boll W., Lindenmann J., Weissmann C. (1986). Mx protein: Constitutive expression in 3T3 cells transformed with cloned Mx cDNA confers selective resistance to influenza virus. Cell.

[201] Larsen R., Røkenes T.P., Robertsen B. (2004). Inhibition of infectious pancreatic necrosis virus replication by Atlantic salmon Mx1 protein. J. Virol..

[202] Caipang C.M.A., Hirono I., Aoki T. (2003). *In vitro* inhibition of fish rhabdoviruses by Japanese flounder, *Paralichthys olivaceus Mx*. Virology.

[203] Skaug B., Chen Z.J. (2010). Emerging role of ISG15 in antiviral immunity. Cell.

[204] Huang X., Huang Y., Cai J., Wei S., Ouyang Z., Qin Q. (2013). Molecular cloning, expression and functional analysis of ISG15 in orange-spotted grouper, *Epinephelus coioides*. Fish Shellfish Immunol..

[205] Langevin C., van der Aa L., Houel A., Torhy C., Briolat V., Lunazzi A., Harmache A., Bremont M., Levraud J.-P., Boudinot P. (2013). Zebrafish ISG15 exerts a strong antiviral activity against RNA and DNA viruses and regulates the interferon response. J. Virol..

[206] Zhang Y.-B., Jiang J., Chen Y.-D., Zhu R., Shi Y., Zhang Q.-Y., Gui J.-F. (2007). The innate immune response to grass carp hemorrhagic virus (GCHV) in cultured *Carassius auratus* blastulae (CAB) cells. Dev. Comp. Immunol..

[207] Sun F., Zhang Y.-B., Jiang J., Wang B., Chen C., Zhang J., Gui J.-F. (2014). GIG1, a novel antiviral effector involved in fish interferon response. Virology.

[208] Sun C., Liu Y., Hu Y., Fan Q., Li W., Yu X., Mao H., Hu C. (2013). GIG1 and GIG2 homologs (CiGIG1 and CiGIG2) from grass carp (*Ctenopharyngodon idella*) display good antiviral activities in an IFN-independent pathway. Dev. Comp. Immunol..

[209] Franchi L., Muñoz-Planillo R., Núñez G. (2012). Sensing and reacting to microbes through the inflammasomes. Nat. Immunol..

[210] Schroder K., Tschopp J. (2010). The inflammasomes. Cell.

[211] Arend W.P., Palmer G., Gabay C. (2008). IL-1, IL-18, and IL-33 families of cytokines. Immunol. Rev..

[212] Secombes C.J., Wang T., Bird S. (2011). The interleukins of fish. Dev. Comp. Immunol..

[213] Wang T., Bird S., Koussounadis A., Holland J.W., Carrington A., Zou J., Secombes C.J. (2009). Identification of a novel IL-1 cytokine family member in teleost fish. J. Immunol..

[214] Bird S., Zou J., Wang T., Munday B., Cunningham C., Secombes C.J. (2002). Evolution of interleukin-1β. Cytokine Growth Factor Rev..

[215] Fujiki K., Shin D.-H., Nakao M., Yano T. (2000). Molecular cloning and expression analysis of carp (*Cyprinus carpio*) interleukin-1β, high affinity immunoglobulin E Fc receptor γ subunit and serum amyloid A. Fish Shellfish Immunol..

[216] Zou J., Grabowski P.S., Cunningham C., Secombes C.J. (1999). Molecular cloning of interleukin 1β from rainbow trout *Oncorhynchus mykiss* reveals no evidence of an ice cut site. Cytokine.

[217] Ingerslev H., Cunningham C., Wergeland H. (2006). Cloning and expression of TNF-α, IL-1β and COX-2 in an anadromous and landlocked strain of Atlantic salmon (*Salmo salar* L.) during the smolting period. Fish Shellfish Immunol..

[218] Pelegrín P., García-Castillo J., Mulero V., Meseguer J. (2001). Interleukin-1β isolated from a marine fish reveals up-regulated expression in macrophages following activation with lipopolysaccharide and lymphokines. Cytokine.

[219] Scapigliati G., Buonocore F., Bird S., Zou J., Pelegrin P., Falasca C., Prugnoli D., Secombes C. (2001). Phylogeny of cytokines: Molecular cloning and expression analysis of sea bass *Dicentrarchus labrax* interleukin-1β. Fish Shellfish Immunol..

[220] Lee D.-S., Hong S.H., Lee H.-J., Jun L.J., Chung J.-K., Kim K.H., do Jeong H. (2006). Molecular cDNA cloning and analysis of the organization and expression of the IL-1β gene in the Nile tilapia, *Oreochromis niloticus*. Comp. Biochem. Physiol. A.

[221] Lu D.-Q., Bei J.-X., Feng L.-N., Zhang Y., Liu X.-C., Wang L., Chen J.-L., Lin H.-R. (2008). Interleukin-1β gene in orange-spotted grouper, *Epinephelus coioides*: Molecular cloning, expression, biological activities and signal transduction. Mol. Immunol..

[222] Covello J., Bird S., Morrison R., Battaglene S., Secombes C., Nowak B. (2009). Cloning and expression analysis of three striped trumpeter (*Latris lineata*) pro-inflammatory cytokines, TNF-α, IL-1β and IL-8, in response to infection by the ectoparasitic, *Chondracanthus goldsmidi*. Fish Shellfish Immunol..

[223] Corripio-Miyar Y., Bird S., Tsamopoulos K., Secombes C. (2007). Cloning and expression analysis of two pro-inflammatory cytokines, IL-1β and IL-8, in haddock (*Melanogrammus aeglefinus*). Mol. Immunol..

[224] Tsutsui S., Iwamoto T., Nakamura O., Watanabe T. (2007). LPS induces gene expression of interleukin-1β in conger eel (*Conger myriaster*) macrophages: First cytokine sequence within anguilliformes. Fish Shellfish Immunol..

[225] Pleguezuelos O., Zou J., Cunningham C., Secombes C.J. (2000). Cloning, sequencing, and analysis of expression of a second IL-1β gene in rainbow trout (*Oncorhynchus mykiss*). Immunogenetics.

[226] Engelsma M.Y., Stet R.J., Saeij J.P., Verburg-van Kemenade B.L. (2003). Differential expression and haplotypic variation of two interleukin-1β genes in the common carp (*Cyprinus carpio* L.). Cytokine.

[227] Husain M., Bird S., van Zwieten R., Secombes C.J., Wang T. (2012). Cloning of the IL-1β3 gene and IL-1β4 pseudogene in salmonids uncovers a second type of IL-1β gene in teleost fish. Dev. Comp. Immunol..

[228] Wang Y., Wang Q., Baoprasertkul P., Peatman E., Liu Z. (2006). Genomic organization, gene duplication, and expression analysis of interleukin-1β in channel catfish (*Ictalurus punctatus*). Mol. Immunol..

[229] Hong S., Zou J., Collet B., Bols N.C., Secombes C.J. (2004). Analysis and characterisation of IL-1β processing in rainbow trout, *Oncorhynchus mykiss*. Fish Shellfish Immunol..

[230] Pelegrín P., Chaves-Pozo E., Mulero V., Meseguer J. (2004). Production and mechanism of secretion of interleukin-1β from the marine fish gilthead seabream. Dev. Comp. Immunol..

[231] Koussounadis A.I., Ritchie D.W., Kemp G.J., Secombes C.J. (2004). Analysis of fish IL-1β and derived peptide sequences indicates conserved structures with species-specific IL-1 receptor binding: Implications for pharmacological design. Curr. Pharm. Des..

[232] Bilen S., Biswas G., Otsuyama S., Kono T., Sakai M., Hikima J.-I. (2014). Inflammatory responses in the Japanese pufferfish (*Takifugu rubripes*) head kidney cells stimulated with an inflammasome-inducing agent, nigericin. Dev. Comp. Immunol..

[233] Martin S.A., Zou J., Houlihan D.F., Secombes C.J. (2007). Directional responses following recombinant cytokine stimulation of rainbow trout (*Oncorhynchus mykiss*) RTS-11 macrophage cells as revealed by transcriptome profiling. BMC Genomics.

[234] Yin Z., Kwang J. (2000). Carp interleukin-1β in the role of an immuno-adjuvant. Fish Shellfish Immunol..

[235] Hong S., Peddie S., Campos-Pérez J.J., Zou J., Secombes C.J. (2003). The effect of intraperitoneally administered recombinant IL-1β on immune parameters and resistance to *Aeromonas salmonicid*a in the rainbow trout (*Oncorhynchus mykiss*). Dev. Comp. Immunol..

[236] Angosto D., López-Castejón G., López-Muñoz A., Sepulcre M.P., Arizcun M., Meseguer J., Mulero V. (2012). Evolution of inflammasome functions in vertebrates: Inflammasome and caspase-1 trigger fish macrophage cell death but are dispensable for the processing of IL-1β. Innate Immun..

[237] Reis M.I., do Vale A., Pereira P.J., Azevedo J.E., dos Santos N.M. (2012). Caspase-1 and IL-1β processing in a teleost fish. PLoS ONE.

[238] Vojtech L.N., Scharping N., Woodson J.C., Hansen J.D. (2012). Roles of inflammatory caspases during processing of zebrafish interleukin-1β in *Francisella noatunensis* infection. Infect. Immun..

